# Electroactive Covalent Organic Framework Enabling Photostimulus-Responsive Devices

**DOI:** 10.1021/jacs.2c06333

**Published:** 2022-08-25

**Authors:** Yizhou Yang, Amritha P Sandra, Alexander Idström, Clara Schäfer, Martin Andersson, Lars Evenäs, Karl Börjesson

**Affiliations:** † Department of Chemistry and Molecular Biology, University of Gothenburg, 41296 Gothenburg, Sweden; ‡ Department of Chemistry and Chemical Engineering, Chalmers University of Technology, 41296 Gothenburg, Sweden

## Abstract

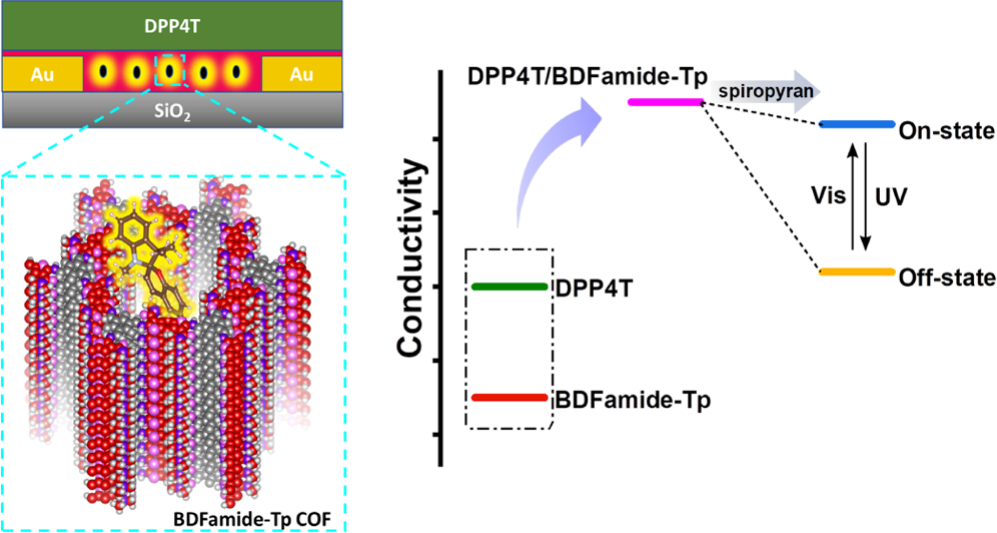

Two-dimensional covalent organic frameworks (2D COFs) feature graphene-type 2D layered sheets but with a tunable structure, electroactivity, and high porosity. If these traits are well-combined, then 2D COFs can be applied in electronics to realize functions with a high degree of complexity. Here, a highly crystalline electroactive COF, BDFamide-Tp, was designed and synthesized. It shows regularly distributed pores with a width of 1.35 nm. Smooth and successive films of such a COF were fabricated and found to be able to increase the conductivity of an organic semiconductor by 10^3^ by interfacial doping. Upon encapsulation of a photoswitchable molecule (spiropyran) into the voids of the COF layer, the resulted devices respond differently to light of different wavelengths. Specifically, the current output ratio after UV vs Vis illumination reaches 100 times, thus effectively creating on and off states. The respective positive and negative feedbacks are memorized by the device and can be reprogrammed by UV/Vis illumination. The reversible photostimulus responsivity and reliable memory of the device are derived from the combination of electroactivity and porosity of the 2D COF. This work shows the capability of 2D COFs in higher-level electronic functions and extends their possible applications in information storage.

## Introduction

Covalent organic frameworks (COFs) are known as predesigned two- or three-dimensional network structures with an atomic-precise distribution of atoms and pores.^1,2^ An increasing number of COFs with abundant structural diversities are actively being synthesized and explored in a wide range of applications, including separation,^3−7^ catalysis,^8−13^ optoelectronics,^14−19^ energy storage,^20−23^ etc. Among these, 2D COFs attract particular interest in the field of organic electronics because of their high crystallinity, structural tunability, and their graphene-like 2D layered features.^24−26^ Electroactive COFs are investigated from multiple design strategies, for instance, by introducing known electroactive building blocks into the frameworks or by focusing on creating full conjugation over the network.^27,28^ Electroactive COFs are actively applied in various devices such as photodetectors,^29−31^ field-effect transistors,^32−34^ and electrochromic devices.^35,36^ The exploration of new chemical structures to improve electroactivity receives much attention, yet the other talent of COFs, their large porosity, is comparatively neglected in device applications. As it is difficult to find porous structures within traditional organic electronic materials, it is interesting to use the porosity of an electroactive COF to create functions that traditional materials have difficulties to perform.

Photostimulus-responsive devices often serve as the central unit in optoelectronics, such as photodetectors,^37^ photoswitches, and photomemory-based storages.^38−41^ By incorporating photoactive building blocks into the framework structure, electroactive COFs can be endowed with photostimulus responsivity.^42^ This photostimulus responsivity of the device is mainly based on two kinds of mechanisms, the photoelectric effect and the photoswitching effect. In the photoelectric mechanism, a strong light-absorbing unit in the COF structure absorbs photons, which results in a photocurrent as an output signal. High sensitivity can be achieved, enabling functions such as photodetectors. However, no photostimulus memory is possible, and only real-time responsivity can be achieved. Furthermore, devices cannot recognize light with different wavelengths. This is because light absorption from all wavelengths gives a positive current gain.^43,44^

In the photoswitching mechanism, the photoswitchable unit in the COF structure changes its chemical configuration. This leads to different charge transfer abilities, recorded as increased/decreased conductivity.^45^ In this case, the stimulus can be memorized, and different wavelengths can be distinguished by positive/negative feedback. Zhang et al. innovatively introduced dithienylethene ligands into a COF structure and realized photoswitchable conductivity when switched by UV/Vis light.^45^ However, after this demonstration, no further reported devices based on photoswitchable COFs exist. This is most likely because the incorporation of a photoswitchable molecule into a COF structure requires compatibility between the photoswitch structure and the COF synthetic method.

Herein, we present a strategy to realize a photostimulus-responsive device by incorporating photoswitchable molecules into the pores of an electroactive COF. The benefit of incorporating the photoswitchable unit into the pores of the COF is that any commercially available photoswitch of compatible size can be incorporated in this manner.^46^ A 2D COF, BDFamide-Tp, featuring an electroactive benzodifuran moiety was synthesized and used as a co-active layer in a thin film device. The COF layer significantly improves the conductivity of a conjugated polymer by 3 orders of magnitude. By encapsulating a photoswitchable spiropyran molecule into the pores of the COF, the devices give stable negative/positive feedback of 2 orders of magnitude when illuminated with UV/Vis light. The mechanism enabling the high performance includes (i) interfacial doping by the electroactive COF to improve the conductivity of a conjugated polymer, providing a wide performance window for the device, (ii) a large porosity of the COF, enabling encapsulation of photoswitchable molecules in the functional layer, and (iii) two metastable states (spiropyran and merocyanine) that can modulate interfacial doping.

## Results and Discussion

### Synthesis of BDF-Dicarboxamide and BDFamide-Tp

**Figure 1:**
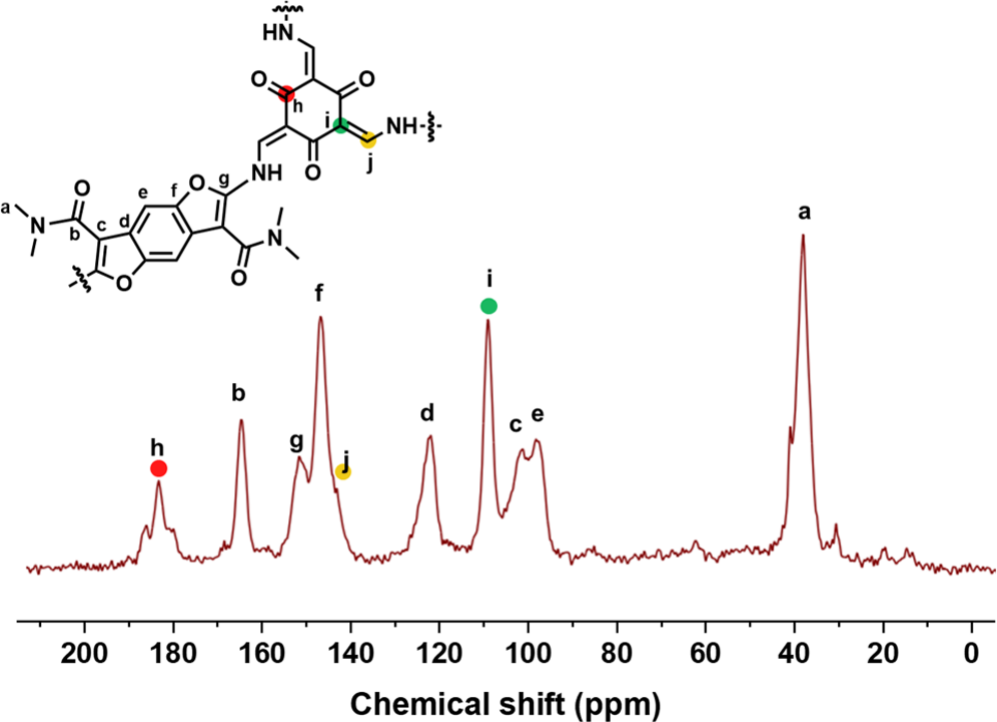
^13^C CP/MAS solid-state NMR spectrum of the COF BDFamide-Tp.

**Scheme 1:**
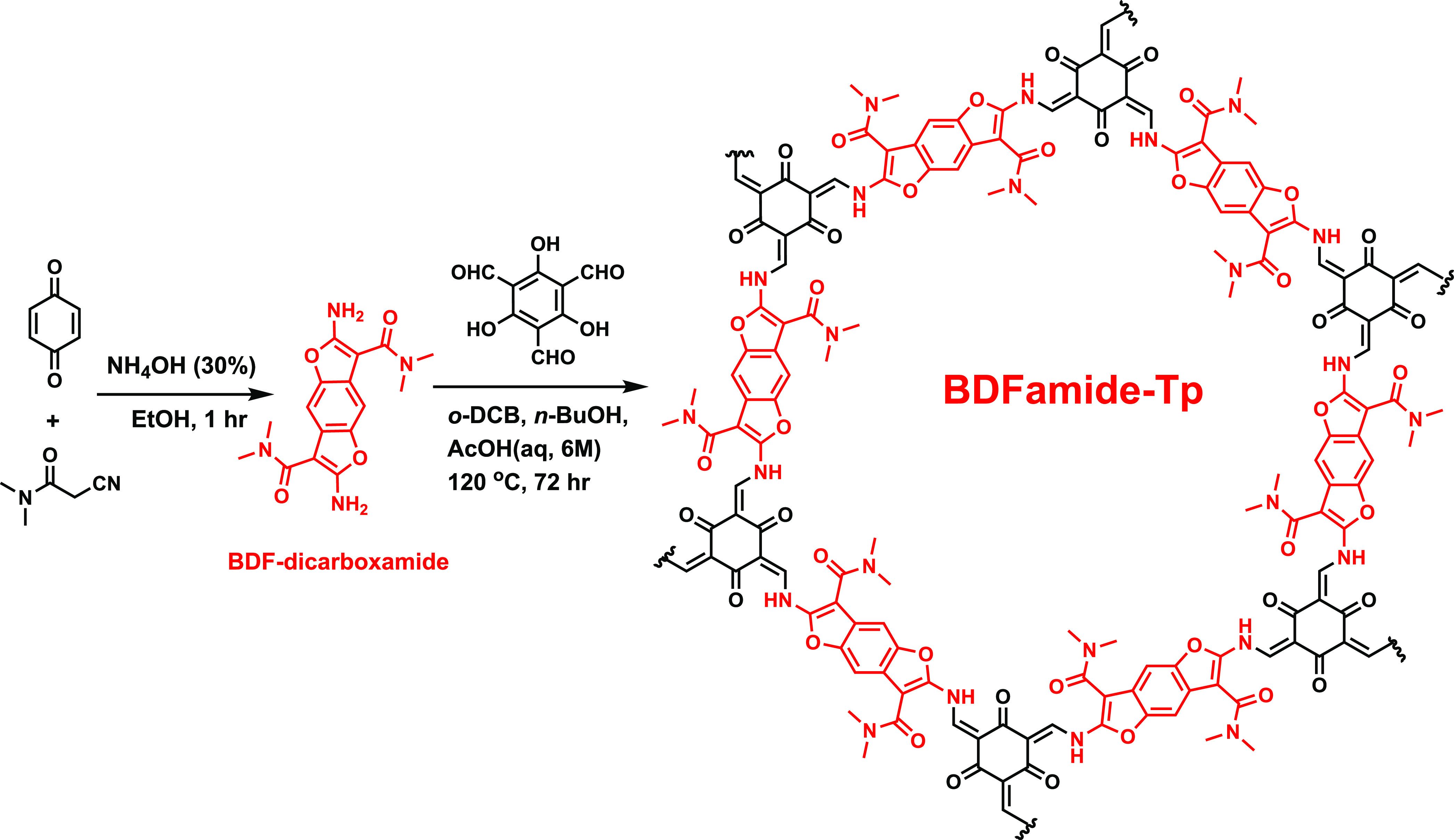
Synthesis of a Two-Dimensional COF BDFamide-Tp

To synthesize an electroactive COF, the main building block, 2,6-diamino-*N*^3^,*N*^3^,*N*^7^,*N*^7^-tetramethylbenzo[1,2-*b*:4,5-*b*′]difuran-3,7-dicarboxamide (BDF-dicarboxamide), was designed (Scheme 1). The conjugated benzodifuran core and dual carboxamide side chains form an acceptor–donor–acceptor system, which is endcapped with amine groups for imine-type dynamic covalent chemistry (Figures S1 and S2). BDF-dicarboxamide was synthesized via a modified literature procedure in a one-pot reaction by a Michael addition between 1,4-benzoquinone and the carbanion of 2-cyano-*N*,*N*-dimethylacetamide, followed by intramolecular double cyclization.^47^ The 2D COF, BDFamide-Tp, was obtained using solvothermal conditions. Under catalysis of acetic acid, the monomers BDF-dicarboxamide and 1,3,5-triformylphloroglucinol (Tp) reacted via an imine-type polymerization, followed by a tautomerization from the product in enol form to the thermodynamically favored keto form BDFamide-Tp (Figure S3). The chemical structure of BDFamide-Tp was confirmed by solid-state ^13^C cross-polarization magic-angle-spinning nuclear magnetic resonance (CP/MAS NMR) analysis (Figure 1). Peaks at 184, 146, and 109 ppm correspond to carbonyl carbon (C=O), enamine carbon (−C–N), and exocyclic carbon (C=C), respectively.^48−50^ These signals are typical for β-ketoenamine type knots formed by tautomerism from an enol to a keto structure. Also, the successful polymerization was verified by FTIR (Figure S4). Peaks at 1240 and 1575 cm^–1^ in the prepared BDFamide-Tp COF represent vibrations of the newly formed C–N and C=C bonds in the β-ketoenamine moiety.^51−53^ In BDFamide-Tp, neither vibrations corresponding to aldehydes (at 1697 cm^–1^ in Tp) nor vibrations corresponding to primary amines (at 3352, 3300, and 3132 cm^–1^ in BDF-dicarboxymide) are present, thus indicating a high conversion of the starting materials. The chemical structure characterizations illustrate the successful condensation from the monomers to a fully polymerized keto-type BDFamide-Tp network.

### Crystallinity and Porosity of BDFamide-Tp

**Figure 2:**
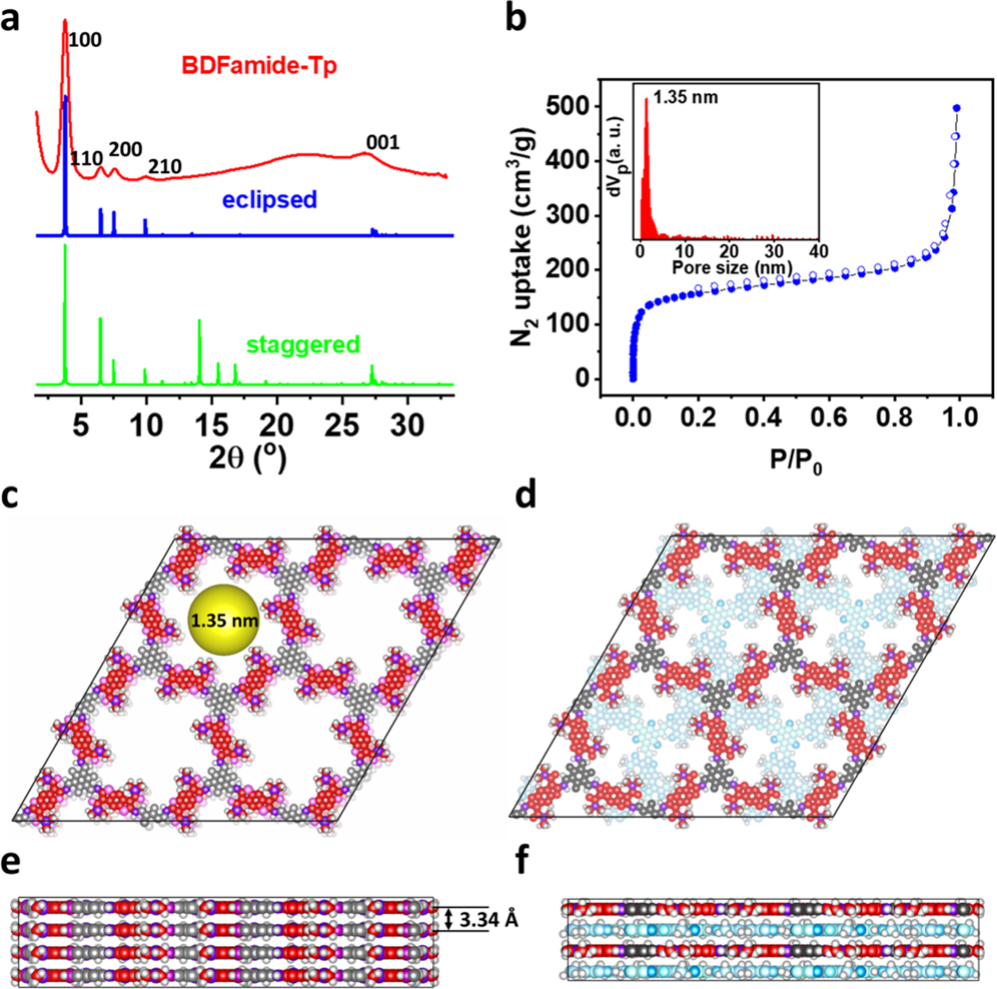
(a) PXRD pattern (red line) of BDFamide-Tp made by solvothermal synthesis and simulated PXRD signal of BDFamide-Tp in an eclipsed (blue line) and staggered (green line) stacking mode. (b) N_2_ adsorption–desorption isotherm of BDFamide-Tp with inset presenting the pore size distribution. (c, e) Top view (c) and side view (e) of BDFamide-Tp in eclipsed stacking mode. The yellow area in panel (c) illustrates the pore size of the structure. (d, f) Top view (d) and side view (f) of BDFamide-Tp in a staggered stacking mode.

To investigate the crystallinity and porosity of BDFamide-Tp, powder X-ray diffraction (PXRD) and N_2_ adsorption–desorption were carried out. As shown in Figure 2a, the PXRD pattern of BDFamide-Tp features a series of clear diffractions, indicating a highly crystalline state. From measured diffraction peak positions combined with structural simulation (Tables S1 and S2), the precise crystal structure can be determined. The peaks at an angle of 3.8, 6.5, 7.6, and 10.0° are attributed to diffraction signals from the (100), (110), (200), and (210) crystal facets, respectively. At a higher angle, the peak at 26.7° represents the (001) facet and indicates an interlayer π–π stacking distance of 3.3 Å, which is a reasonable value for π–π stacking systems.^54,55^ To compare with theoretical PXRD patterns, BDFamide-Tp in eclipsed stacking mode (Figure 2c,e) and staggered stacking mode (Figure 2d,f) was simulated. It can be observed that the experimental data fits with the simulated PXRD of an eclipsed packing mode. Especially the absence of peaks around 15° clearly exclude the probability of a staggered packing mode. Thus, the crystal structure of the 2D COF BDFamide-Tp is revealed to have *hcb*-type topology with the network stacking in an eclipsed mode, forming cylinder channels with nanometer diameters through the out-of-plane direction. The crystal was assigned to the *P*1 space group, with unit cell parameters of *a* = *b* = 26.96 Å, *c* = 3.34 Å, α = β = 90°, and γ = 120°. The porosity of BDFamide-Tp was determined by an N_2_ sorption isotherm at 77 K. The reversible type-I isotherm (Figure 2b) demonstrates the microporous feature of the material, with a Brunauer–Emmett–Teller (BET) surface area of 588 m^2^ g^–1^. The pore size distribution was calculated by nonlocal density functional theory (NLDFT) analysis, presenting a pore width of 1.35 nm.

**Figure 3:**
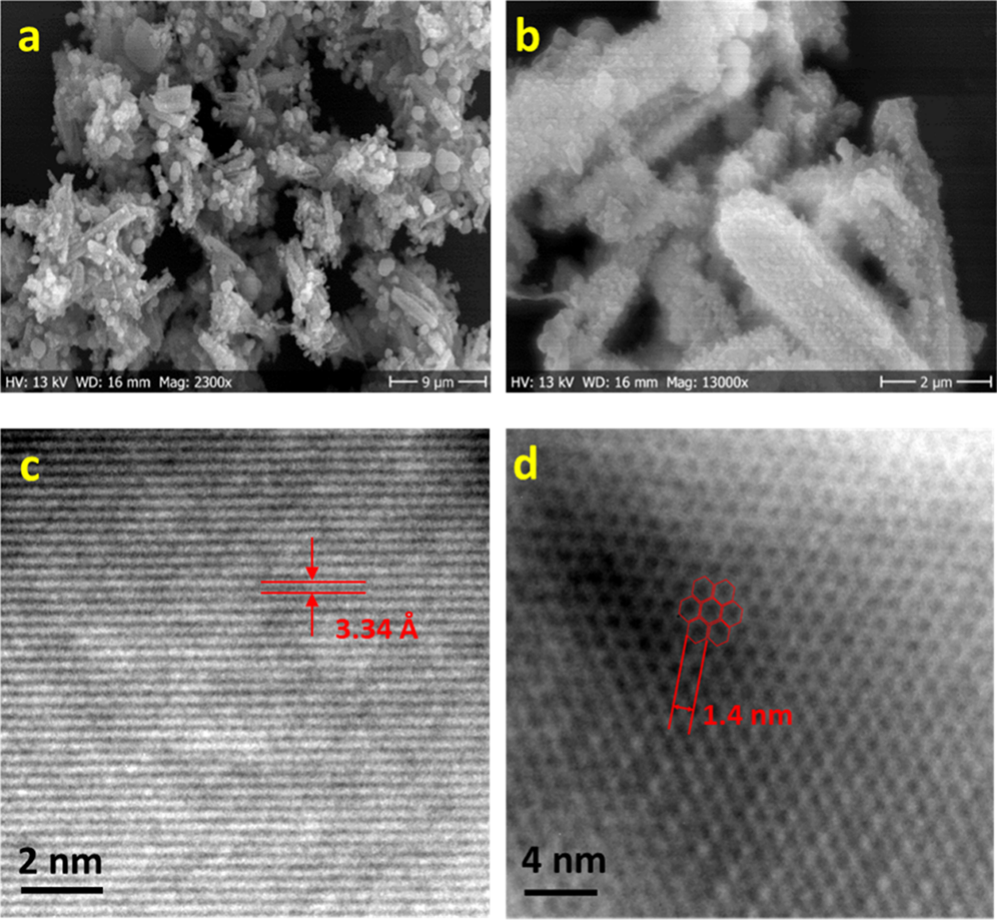
(a, b) SEM images of BDFamide-Tp made by solvothermal synthesis. (c, d) HRTEM images of BDFamide-Tp made by solvothermal synthesis.

Scanning electron microscopy (SEM) and transmission electron microscopy (TEM) were applied to observe the microscopic morphology and nanoscopic structure of BDFamide-Tp. As shown in Figure 3a, the BDFamide-Tp powder exhibits a morphology of micrometer-sized, rod-shaped, and aggregated clusters. The lengths of the rods can reach 7–9 μm. In higher magnification (Figure 3b), more details are revealed. The diameter of the rods is around 0.5–1 μm. Their surfaces are covered with nanoscaled protrusions (Figure S5). Under TEM, most of the area in the material was clearly observed to have a fully ordered lattice texture (Figure S6), presenting a high crystallinity in accordance with PXRD. The different orientations of the lattice domains illustrate a general polycrystallinity of the BDFamide-Tp powder. Interestingly, in some areas, a single lattice orientation can extend through the whole TEM observation window, thus indicating monocrystalline domain sizes of at least several tens of nanometers. The end of a protrusion is shown in Figure S7. A crystal with a regular shape is seen, featuring a lattice of densely stacked lines throughout the whole crystal. From high-resolution TEM (HRTEM) (Figure 3c), the lattice distance was measured to be 3.3 Å, which corresponds to the interlayer stacking distance of 2D layers in BDFamide-Tp, indicating that the (001) crystal facet is observed in Figures 3c and S7. Orderly organized pores with a size of ∼1.4 nm were also observed (Figure 3d), which corroborates the measured pore size from gas adsorption experiments.

### BDFamide-Tp Films

**Figure 4:**
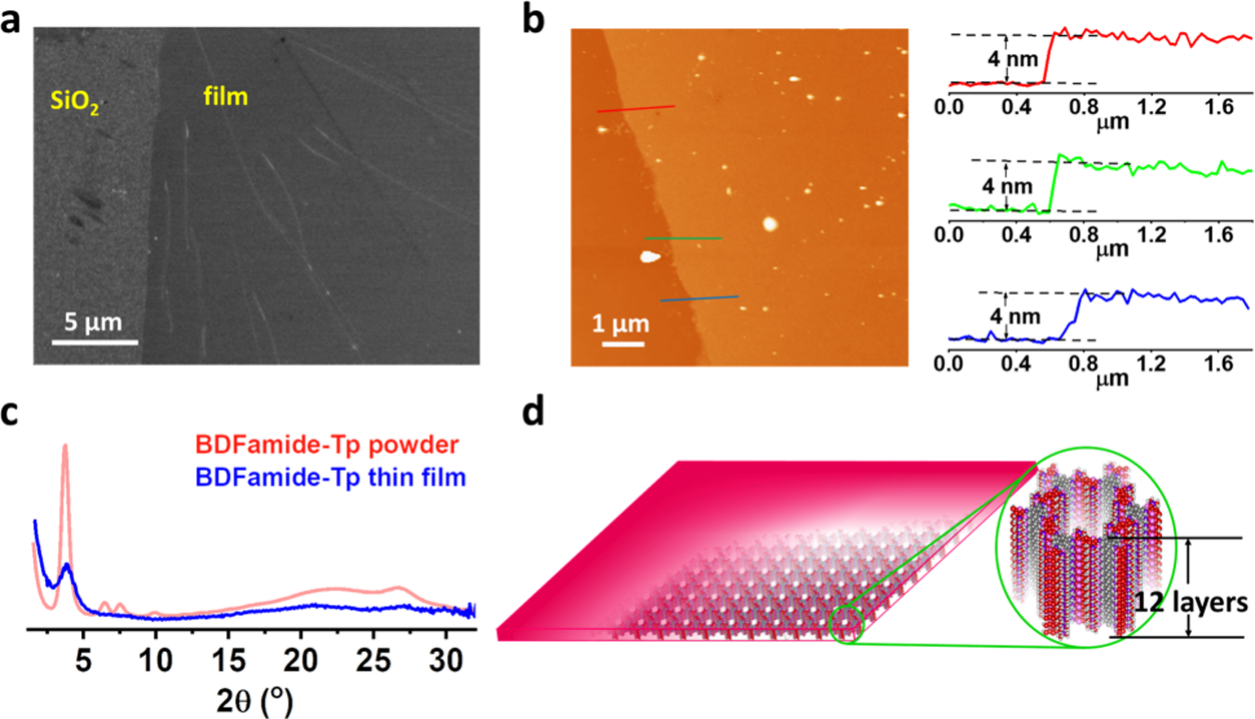
(a) SEM image of a BDFamide-Tp film fabricated by interfacial synthesis. (b) AFM height image of the BDFamide-Tp film (left) and cross-sectional profiles (right) extracted from the AFM height image, showing the thicknesses of the film. (c) GIXRD pattern of a BDFamide-Tp film (blue) compared with the PXRD pattern of BDFamide-Tp powder (red) made from solvothermal synthesis. (d) Illustration of the BDFamide-Tp film showing the thickness in a molecular scale.

For electroactive COF materials to be applied in electronic thin film devices, they must be prepared in a film state.^56^ The continuity and uniformity of the COF film have a significant influence on the performance and stability of the device.^27^ Here, BDFamide-Tp COF films were prepared using interfacial synthesis (Figure S8), which is a widely reported method for the fabrication of COF films.^57−63^ After optimization of synthetic conditions (Table S3 and Figure S9), a BDFamide-Tp film with high quality was obtained at the liquid–liquid interface. Synthesized films of several millimeters in size can be transferred to desired substrates without rupturing for further characterization and device construction. Figures 4a and S10 show SEM images of a BDFamide-Tp film on a SiO_2_ substrate. The film shows a high continuity and a uniform surface, except for some wrinkles on the film. To give a quantitative analysis of the surface morphology, atomic force microscopy (AFM) was performed (Figures 4b and S11). Figure 4b shows an AFM scan at the edge of the BDFamide-Tp COF film, presenting a considerable homogenous morphology. No observable cracks or pinholes are seen, illustrating an internal continuity of the material. The film thickness measured at different sites gives an identical value of 4 nm, indicating a uniform thickness of the BDFamide-Tp COF film. The overall statistical analysis of the AFM height image gives an RMS surface roughness of 0.35 nm, corroborating a high surface smoothness.

It is natural to check if the BDFamide-Tp film prepared via an interfacial method has the same crystalline 2D COF features as the powdered COF made from solvothermal synthesis. Grazing incidence X-ray diffraction (GIXRD) was performed on the film (Figure 4c). Comparing the results to the PXRD pattern from the powder made by solvothermal synthesis, the main diffraction from the film is almost identical in peak position but with less intensity, thus indicating that the internal structure of the film is the same as in the powder. The low signal-noise ratio in GIXRD is due to the ultrathinness of 4 nm of the film. Thus, the homogeneous, smooth, and continuous film is revealed to have an internal ordered 2D layered honeycomb network. According to the thickness of the film and the interlayer spacing, the prepared film contains roughly 12 layers of 2D networks (Figure 4d).

### Photostimulus-Responsive Device

**Figure 5:**
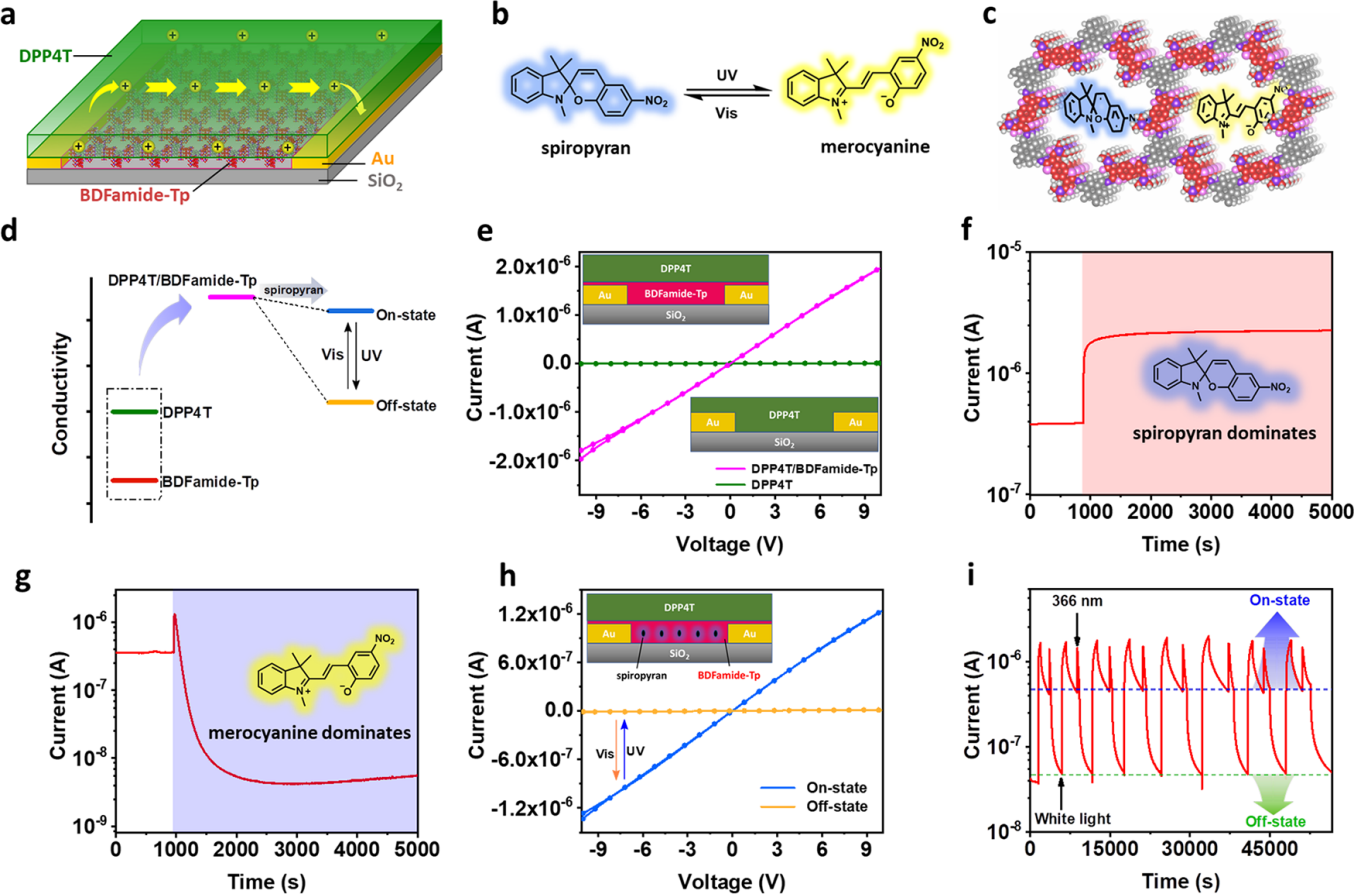
(a) Structural illustration of a thin film device with BDFamide-Tp/DPP4T as an active layer. Gold was used as electrodes and SiO_2_ was used as an insulating substrate. (b) Photoswitch couple showing reversible structure transformation under UV/Vis illumination. (c) Illustration of encapsulated spiropyran/merocyanine in the pores of the BDFamide-Tp COF. (d) Conductivity diagram of the working mechanism of the photoswitchable device. (e) *I*–*V* curves of devices with only DPP4T as an active layer and DPP4T/BDFamide-Tp as an active layer. Each scan includes a forward and a reversed voltage sweep. The insets show a configuration of respective devices. (f) Dynamic current record of the switch-on process of the photoswitchable device. The pink zone indicates exposure of visible light. (g) Dynamic current record of the switch-off process. The purple zone indicates exposure of UV light. (h) *I*–*V* curves of a photoswitchable device after 8 min of UV illumination (yellow, off-state) or 6 min of Vis illumination (blue, on-state). The inset shows the configuration of the photoswitchable device. (i) Current output of the photoswitching device under successive switching on and off operation.

The uniformity, continuity, and transferability of the BDFamide-Tp COF film enable applications for electronic devices. The basic COF film conductivity was measured by transferring the BDFamide-Tp film onto a silicon wafer with a dielectric SiO_2_ surface and predeposited gold electrodes (see Figure S12 for a device structure). A rather weak current at noise level (below 0.1 nA) was detected when a biased sweeping voltage was applied between the electrodes, indicating an insulating property of BDFamide-Tp. The low conductivity of BDFamide-Tp is due to the β-ketoenamine type linkage, which breaks the conjugation between the benzodifuran units. Thus, the band-like charge transfer in the conjugation in the 2D layer is blocked. When an organic semiconductor, DPP4T (see Figure S13), was further deposited above the BDFamide-Tp film, a significant current appeared in the conduction channel as indicated by a linear *I*–*V* curve with negligible hysteresis (see Figure 5a for the device and Figure 5e for *I*–*V* curves). The *I*–*V* curve of pure DPP4T is also displayed in Figure 5e for comparison, which shows a negligible current. It should be mentioned that DPP4T is not conductive when there is no gate voltage applied (as performed in a field-effect transistor).^64^ Therefore, the conductivity of DPP4T is improved by 3 orders of magnitude with BDFamide-Tp underneath, which can be ascribed to an interfacial doping effect.^65−67^ Interfacial doping can occur by proton and/or charge transfer. In this case, the p-type semiconductor DPP4T can transfer electrons to the electron-deficient BDFamide-Tp and produce holes as charge carriers. Based on previously examined P3HT systems,^68,69^ we propose a mechanism for the process (Figure S14).

With this interesting doping phenomenon at hand, a photostimulus-responsive device can be realized if the BDFamide-Tp doping ability can be modulated by light. The idea is to encapsulate photoswitchable molecules into the voids of the COF layer to affect proton/charge transfer within the conductive channels of the device, thus resulting in a low and high conduction state. Here, a commercially available spiropyran was used, which has two states that can be reversibly switched by ultraviolet and visible light (Figures 5b and S15). The neutral spiropyran state should have less influence on the channel current of the device, and the zwitterionic merocyanine state can cause serious perturbations to proton/charge transfer (Figure S16). In addition, the molecular size of the used spiropyran/merocyanine couple is in the same order as the voids of BDFamide-Tp (Figures 5c and S17), therefore enabling encapsulation. To confirm a successful encapsulation, a UV–Vis study was performed. It showed that spiropyran was encapsulated in the BDFamide-Tp film at a concentration of 0.065 M (Figure S18), exhibiting considerable stability and photoswitchability (Figures S19 and S20). FTIR was used as a complement to the UV–Vis data, and it confirmed a successful encapsulation (Figure S21). The mechanism for the designed photostimulus-responsive device is described in Figure 5d. DPP4T and BDFamide-Tp cooperatively reach a high conductive state, which then is split into two states, one in the presence of spiropyran (on-state) and another in the presence of merocyanine (off-state). To mechanistically investigate the dynamic photoswitching process, the channel current was constantly recorded during the switching-on and switching-off operation. The starting state was chosen as the fabricated pristine device, with the spiropyran–merocyanine couple in thermodynamic equilibrium under ambient conditions. When exposed to a constant illumination of visible light, the current increases instantly to reach a stable plateau, which is ascribed to the conversion of merocyanine to spiropyran (Figure 5f) in combination with the photoelectric effect. In contrast, when exposed to constant illumination of UV light, the current undergoes a sharp increase due to the photoelectric effect, followed by an immediate current drop until reaching a plateau, which indicates the conversion from spiropyran to merocyanine (Figure 5g). This shows that the device responds differently and gives the opposite feedback, according to the stimuli from light with different wavelengths, notwithstanding the presence of the photoelectric effect. It should be mentioned that the device is excluded from possible influence caused by the emission of merocyanine because the fluorescence of merocyanine is quenched in the system (Figure S22). Thus, encapsulated spiropyran/merocyanine in the pores of BDFamide-Tp COF adds a recognition ability of light with different wavelengths to the device response. As a control experiment, a device without the encapsulated photoswitchable molecule was also studied during light exposure. For such a device, the *I*–*V* curves show negligible differences before and after the UV/Vis light exposure (Figure S23). Furthermore, both UV and Vis light raise a similar photocurrent increase due to the photoelectric effect, which means that the device is unable to distinguish light having different wavelengths (Figure S24).

The fabricated photoswitchable device shows very different conductivities as illustrated by *I*–*V* curves (Figures 5h and S25) when switching between the off and on states by UV/Vis light. The output current in the on and off states can reach 1.2 × 10^–6^ A (*V* = 10 V) and 1.2 × 10^–8^ (*V* = 10 V), respectively. This results in a conductivity ratio of 2 orders of magnitudes between the on and off states. The two states of the device were switched for 30 cycles (Figure S26), showing good operational reproducibility. The output current was also recorded upon successive on–off switching, which displays stable on and off currents between cycles (Figure 5i), further showing the reliable device performance.

## Conclusions

In summary, we have designed and successfully synthesized an electroactive COF, BDFamide-Tp, based on the building block BDF-dicarboxamide. The BDFamide-Tp COF shows a high crystallinity with an intrinsically ordered structure of 2D networks with *hcb* topology that are stacked in an eclipsed mode, featuring pores with a width of 1.35 nm. Furthermore, high-quality thin films of BDFamide-Tp were fabricated via interfacial synthesis for electronic applications. Photoswitchable molecules can be encapsulated into the pores of the COF that together with an organic semiconductor can function as a responsive device to give specific feedback, according to different wavelengths of photostimuli. In such devices, the 2D COF BDFamide-Tp plays two important roles for the successful function. First, BDFamide-Tp shows electroactivity, significantly improving the conductivity of the organic semiconductor DPP4T, enabling a stable current and wide-working window of the device. Second, the porosity of BDFamide-Tp allows the encapsulation of a molecular photoswitch in the active layer, which endows a modulated doping effect when exposed to light with different wavelengths. This work shows a successful example of utilizing both electroactivity and porosity of COFs to realize device functions at a high level of complexity. Thus, COFs provide a new choice for organic electronics, where functionalities can be integrated without disturbing the internal communication of the core structure.

## Supporting Information Available

Materials and methods; experimental detail; supplementary figures; modeling details; NMR spectra (PDF)
