# Supplementary material for: Electroactive Covalent Organic Framework Enabling Photostimulus-Responsive Devices
Source: J Am Chem Soc. 2022 Aug 25;144(35):16093–100. doi: 10.1021/jacs.2c06333 (PMC9460776; doi:10.1021/jacs.2c06333)
Supplement: Supplementary file 1 — ja2c06333_si_001.pdf [file ja2c06333_si_001.pdf]

Supporting Information

Electroactive Covalent Organic Framework Enabling Photo-stimulus-responsive Devices

Yizhou Yang,[1] Amritha P Sandra,[1†] Alexander Idström,[2] Clara Schäfer,[1] Martin Andersson,[2] Lars Evenäs,[2] Karl Börjesson[1]*

[1]Department of Chemistry and Molecular Biology, University of Gothenburg, 41296 Gothenburg, Sweden

[2]Department of Chemistry and Chemical Engineering, Chalmers University of Technology, 41296 Gothenburg, Sweden

*Corresponding Author: karl.borjesson@gu.se

Experimental section

MATERIALS AND METHODS

Chemicals

All starting materials and solvents are purchased from Sigma Aldrich, VWR or TCI Europe and used without further purification. The photoswitchable molecule spiropyran was purchased from Sigma Aldrich. The semiconducting polymer DPP4T was synthesized according to reported literature.[1] Solvothermal synthesis were carried out under nitrogen atmosphere using a standard Schlenk line technique.

Instrumentation

Nuclear magnetic resonance (NMR) for liquid-state analysis was recorded using a Varian 400 spectrometer (400 MHz ^{1}H; 100 MHz ^{13}C) in DMSO-d_6 as solvent. All solid-state NMR measurements were conducted on a Bruker Avance III 500 equipped with a 4 mm HX CP-MAS probe. Measurements were performed at 298 K using a MAS spinning rate of 20 kHz. A cross-polarization magic angle spinning (CP/MAS) pulse sequence with a SPINAL-64 decoupling sequence was used.

Gas adsorption was measured at 77.35 K on a BELSORP-max II analyser for surface area and a N_2 sorption isotherms. BET specific surface area was calculated using ISO 9277 for type I adsorption isotherms. Pore size distribution was obtained by fitting both Gran Canonical Monte Carlo (GCMC) and Non-Localized Density Functional Theory (NLDFT) to the adsorption data. The kernel applied in both cases was for nitrogen as adsorptive, the temperature was 77 K, and the slit-shaped pores of graphitic carbon for the adsorbent geometry. The two methods show good agreement on pore size distribution.

High resolution mass spectroscopy (HRMS) was obtained from an Agilent 1290 Infinity LC system equipped with an autosampler in tandem with an Agilent 6520 Accurate Mass Q-TOF LC/MS.

UV-Vis spectra were measured on a Lambda 950 UV/VIS/NIR spectrometer from PerkinElmer.

Emission spectra were measured on an FLS1000 spectrofluorometer from Edinburgh Instruments.

Fourier transform infrared (FT-IR) were recorded using a Bruker Invenio R instrument in transmission mode.

Scanning Electron Microscopy (SEM) were carried out using a JEOL JSM-6301F scanning electron microscope with an acceleration voltage of 13 kV. The samples were placed on silicon wafers for observation.

Transmission Electron Microscopy (TEM) were carried out using a FEI Tecnai T20 transmission electron microscope with a LaB6 electron source under the acceleration voltage of 200 kV.

Powder X-ray diffraction (PXRD) and **Grazing incidence X-ray diffraction (GIXRD)** data were obtained on a Mat:Nordic SAXSLAB instrument. The detector used was a Pilatus3 300KR from Dectris, and the source was a Rigaku Micromax-003 with a Cu target. All measurements were carried out at a pressure of approximately 0.1 mbar in the entire flightpath. For PXRD, measurements were conducted in transmission mode. The instrument was calibrated by silver behenate before measurement. For GIXRD, the incidence angle was varied between 0.2 and 0.4°, depending on the sample. Before measurement, a calibration of the sample tilt was performed with a z-scan and a rocking curve.

Atomic Force Microscopy (AFM) for film samples were conducted on NT-MDT NTEGRA AFM in tapping mode using a silicon cantilever (Tap150Al-G from Budget Sensors). Silicon wafers were used as substrates.

Electrical characterization was conducted using a Keithley 2600B SMU system at ambient conditions.

Structural modeling

The structural modeling of COFs in different packing modes and spiropyran/merocyanine were generated using BIOVA Material Studio (2017). The geometry and unit cells were optimized using the Forcite method. A general force field and a quasi-Newton algorithm were used in the calculation.

Synthetic details

Synthesis of 2,6-diamino-*N*³,*N*³,*N*⁷,*N*⁷-tetramethylbenzo[1,2-b:4,5-b']difuran-3,7-dicarboxamide (BDF-dicarboxamide)

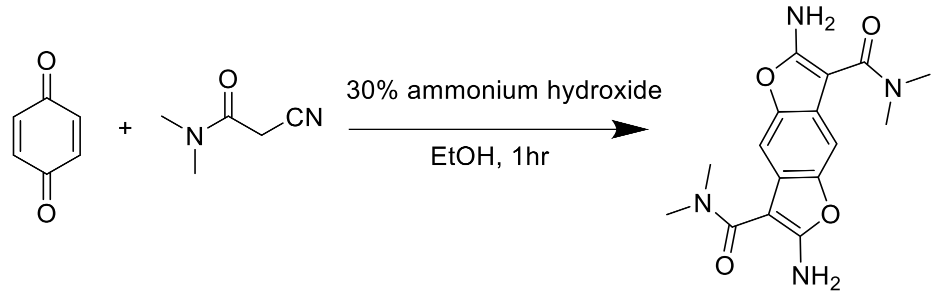

To a solution of 1,4-benzoquinone (1.00 g, 9.26 mmol) dissolved in ethanol (15 mL), 2-cyano-*N*,*N*-dimethylacetamide (2.02 g, 18 mmol) was added at room temperature. Then the addition of 30% ammonium hydroxide (3.5 mL) led to a fast exothermic reaction. The reaction was left to stir for 1 h at room temperature, afterwards, the reaction mixture was centrifuged and washed with ethanol (3 times). After drying under vacuum for 2 days, a greyish white solid was obtained (350 mg, 1.06 mmol, 12%). ^{1}H NMR (400 MHz, DMSO-d6) δ: 7.06 (s, 2H), 6.88 (s, 4H), 2.97 (s, 12H). HRMS: (ESI+) m/z calcd. For $(M+H)^+$ $C_{16}H_{18}N_4O_4$: 331.1406; found: 331.1411.

Synthesis of 2D COF BDFamide-Tp (Solvothermal method)

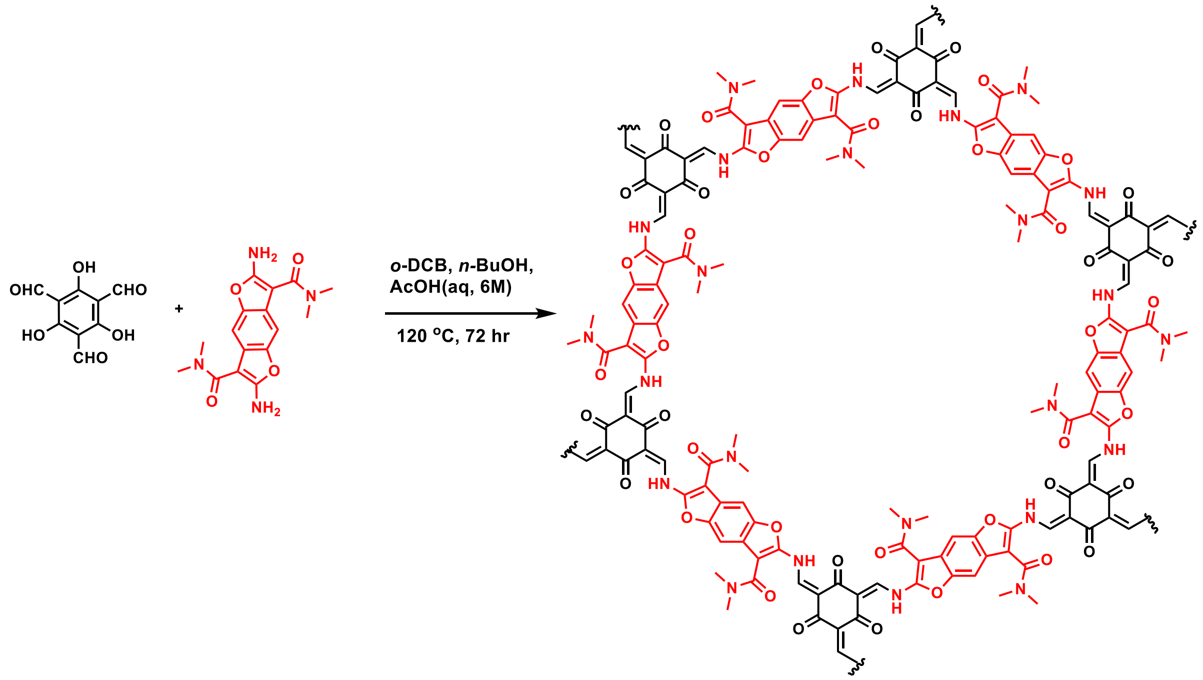

A Schlenk tube was charged with 1,3,5-triformylphloroglucinol (20 mg, 0.09 mmol), BDF-dicarboxamide (44.5 mg, 0.135 mmol), *o*-dichlorobenzene and *n*-butanol (1:1 = v/v, 2.6 mL)

and acetic acid (6 M, 0.135 mL). The mixture was sonicated for 5 minutes, and the tube was degassed three times by freeze-pump-thaw cycles. Then the reaction mixture was heated to 120°C for 72 h. The resulting precipitate was centrifuged and then washed with THF and DMSO several times to get rid of the unreacted reactants and afterwards dried under vacuum (72 h) to remove residual solvents. The product was isolated as a dark brown powder (60 mg, 99 %).

Synthesis of 2D COF BDFamide-Tp film (interfacial method)

The freshly prepared monomer solution BDF-dicarboxamide (1.89×10^{-3} M, in NMP) and Tp (1.25×10^{-3} M, in $CHCl_3$) were sonicated for 10 min for complete dissolution. To a vial (2.5 cm in diameter, 5.5 cm in depth), BDF-dicarboxamide solution and Tp solution were added and mixed to form a uniform solution. Then acetic acid (aq, 6M) was added to the solution and the vial was shaken for 1 min, followed by keeping the vial static to form an immiscible liquid-liquid system. The specific volumes of each solution are shown in Table S3. The systems were placed in a vibration-free environment for one week. BDFamide-Tp films were observed to have formed at the liquid-liquid interface. The as prepared films can be transferred to desired substrates and washed with deionized water, THF and chloroform thoroughly to remove possible residual reactant and byproducts.

Photo-stimulus-responsive device construction and electrical characterization

The BDFamide-Tp COF films were transferred onto silicon wafer chips with predeposited gold electrodes on a 300 nm SiO_2 dielectric surface. The films were washed thoroughly by deionized water, THF, chloroform and isopropanol, and dried by a nitrogen flow. Then the chips were immersed into a spiropyran solution (4 mg/mL, in chloroform) and kept at 50 ^oC for accelerating encapsulation. After 3 days, the chips were taken out and rinsed with chloroform to wash away un-encapsulated spiropyran molecules on the surface. Then, a DPP4T film was deposited on top of the COF layer by spin-coating a DPP4T solution (5 mg/mL, in chloroform) at a speed of 3000 revolutions per minute. The devices were characterized on a Keithley 2600B SMU electrical characterization system at ambient conditions. The switching-on of the devices were realized by irradiation of white light (> 440 nm) for 8 min and the switching-off of the devices were realized by irradiation of 365 nm light for 6 min.

The control device with pure COF/DPP4T as active layer was prepared in the similar way, except for the spiropyran encapsulation step.

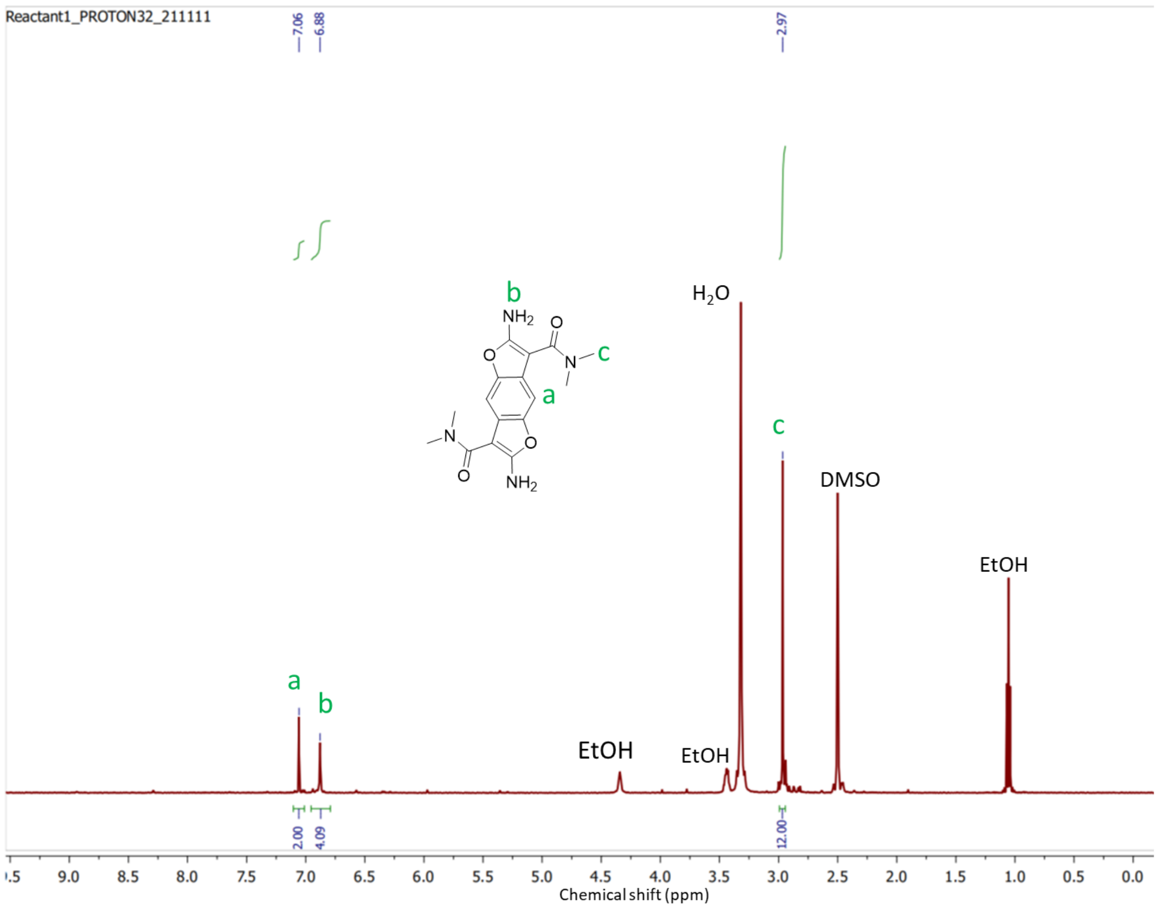

Figure S1. ^{1}H NMR (400 MHz) spectrum of BDF-dicarboxamide in dmso-d6.

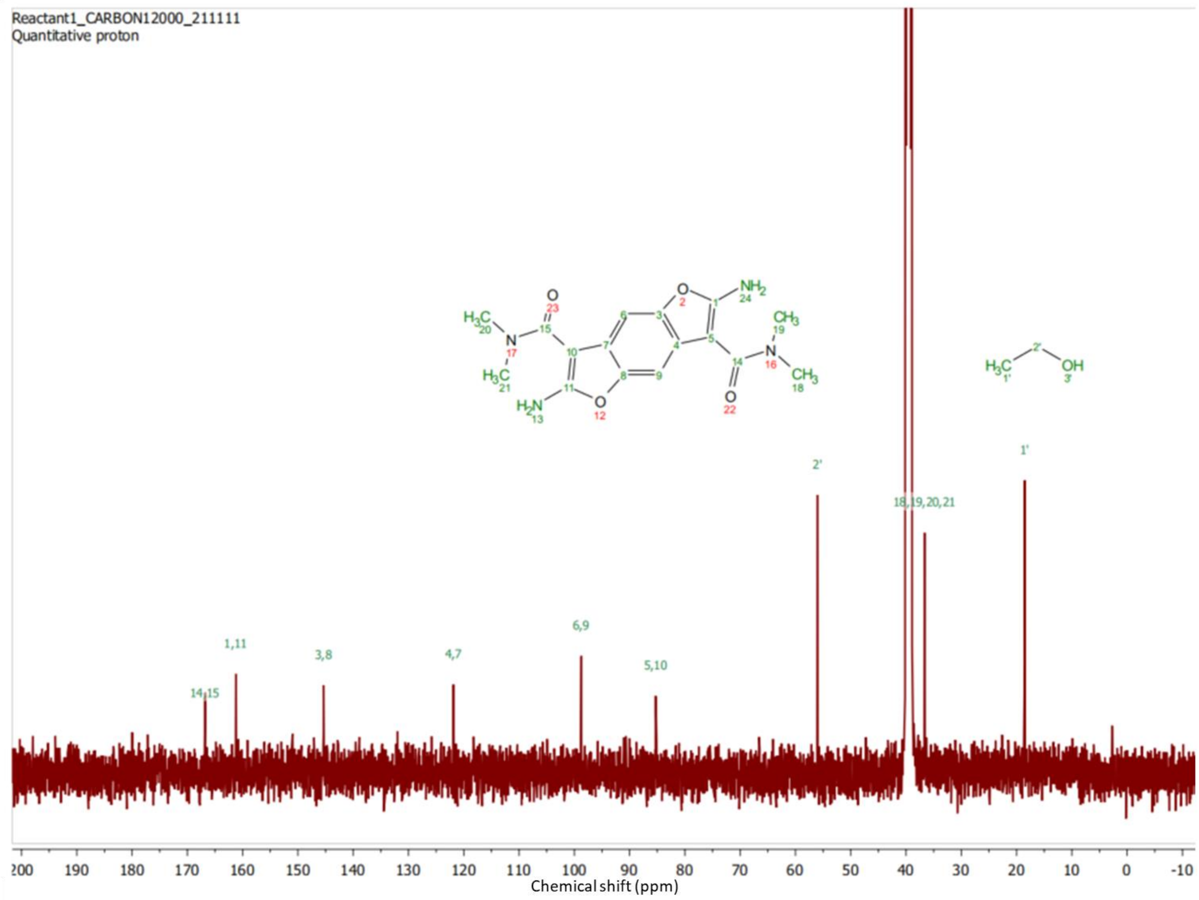

Figure S2. ^{13}C NMR (100 MHz) spectrum of BDF-dicarboxamide in dmso-d6.

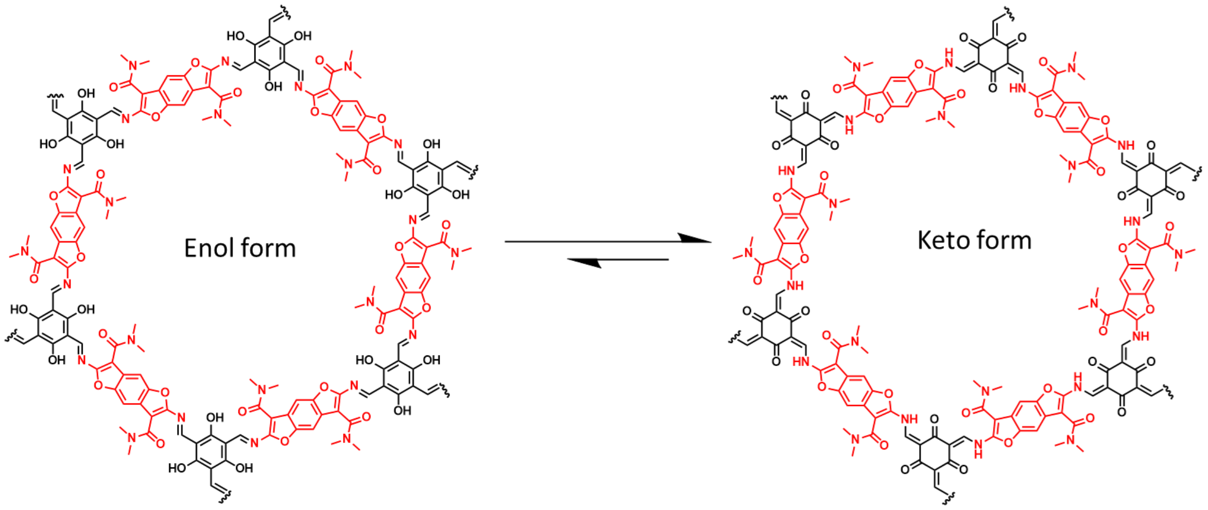

Figure S3. Chemical structure illustrating the tautomerisation of BDFamide-Tp from the enol-form product to the keto-form product.

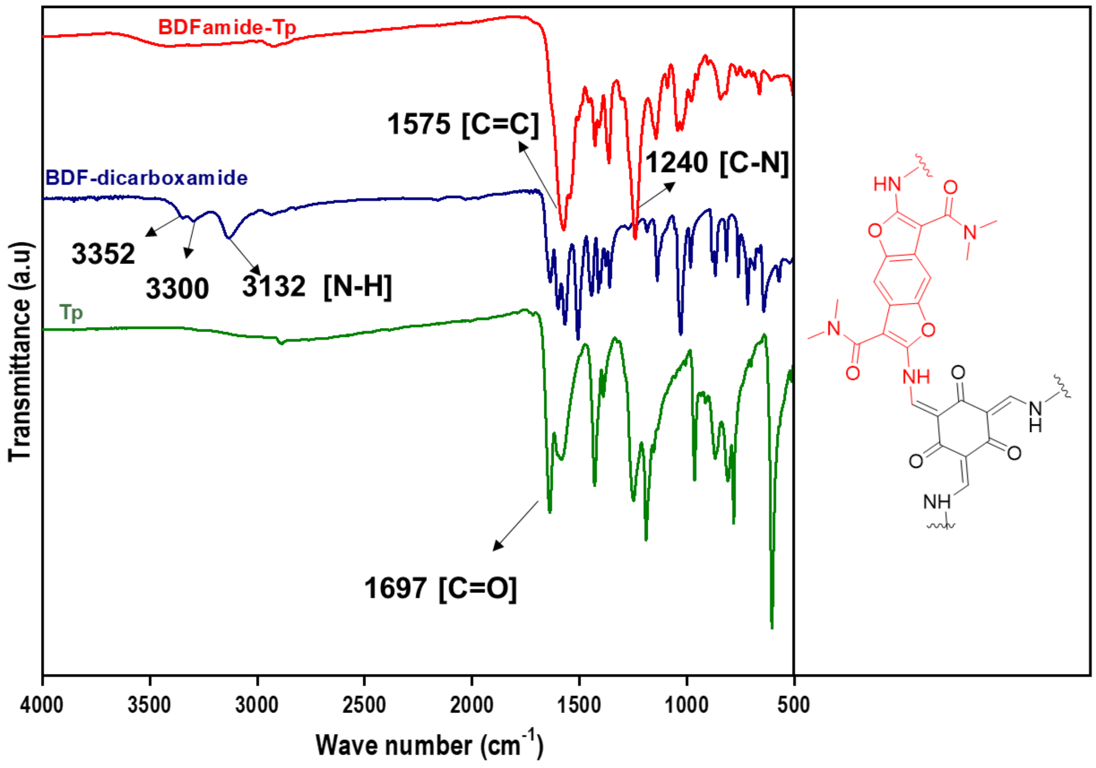

Figure S4. Comparison of FT-IR spectra of BDFamide-Tp with starting material BDF-dicarboxamide and Tp.

Table S1. Fractional atomic coordinates for the unit cell of the BDFamide-Tp COF in eclipsed stacking mode.

BDFamide-Tp in eclipsed stacking mode			
Space group: P-1<br>a = b = 26.9603 Å, c = 3.3405 Å, α =90°, β = 90°, γ = 120°			
Atom	x	y	z
C1	0.30026	0.68955	0.98515
C2	0.27737	0.63359	0.98515
C3	0.39553	0.61662	0.98515
N4	0.38056	0.56805	0.98515
O5	0.29102	0.55795	0.98515
C6	0.40967	0.54204	0.98515
C7	0.46564	0.57511	0.98515
C8	0.49832	0.63668	0.98515
O9	0.47457	0.65989	0.98515
C10	0.57956	0.72324	0.98515
N11	0.55667	0.67127	0.98515
C12	0.59551	0.65632	0.98515
C13	0.48853	0.54204	0.98515
C14	0.44671	0.48852	0.98515
O15	0.39797	0.48852	0.98515
C16	0.45859	0.44805	0.98515
C17	0.31045	0.61071	0.98515
C18	0.36641	0.64378	0.98515
C19	0.38338	0.77891	0.98515
N20	0.43195	0.81251	0.98515
O21	0.44205	0.73307	0.98515
C22	0.45796	0.86763	0.98515
C23	0.42489	0.89053	0.98515
C24	0.36332	0.86164	0.98515
O25	0.34011	0.81468	0.98515
C26	0.27676	0.85632	0.98515
N27	0.32873	0.8854	0.98515
C28	0.34368	0.93919	0.98515
C29	0.45796	0.94649	0.98515
C30	0.51148	0.95819	0.98515
O31	0.51148	0.90945	0.98515
C32	0.55195	0.01054	0.98515
C33	0.38929	0.69974	0.98515
C34	0.35622	0.72263	0.98515
C35	0.22109	0.60447	0.98515
N36	0.18749	0.61944	0.98515
O37	0.26693	0.70898	0.98515
C38	0.13237	0.59033	0.98515
C39	0.10947	0.53436	0.98515
C40	0.13836	0.50168	0.98515
O41	0.18532	0.52543	0.98515
C42	0.14368	0.42044	0.98515
N43	0.1146	0.44333	0.98515
C44	0.06081	0.40449	0.98515
C45	0.05351	0.51147	0.98515
C46	0.04181	0.55329	0.98515
O47	0.09055	0.60203	0.98515
C48	0.98946	0.54141	0.98515
C49	0.69974	0.31045	0.98515
C50	0.72263	0.36641	0.98515

C51	0.60447	0.38338	0.98515
N52	0.61944	0.43195	0.98515
O53	0.70898	0.44205	0.98515
C54	0.59033	0.45796	0.98515
C55	0.53436	0.42489	0.98515
C56	0.50168	0.36332	0.98515
O57	0.52543	0.34011	0.98515
C58	0.42044	0.27676	0.98515
N59	0.44333	0.32873	0.98515
C60	0.40449	0.34368	0.98515
C61	0.51147	0.45796	0.98515
C62	0.55329	0.51148	0.98515
O63	0.60203	0.51148	0.98515
C64	0.54141	0.55195	0.98515
C65	0.68955	0.38929	0.98515
C66	0.63359	0.35622	0.98515
C67	0.61662	0.22109	0.98515
N68	0.56805	0.18749	0.98515
O69	0.55795	0.26693	0.98515
C70	0.54204	0.13237	0.98515
C71	0.57511	0.10947	0.98515
C72	0.63668	0.13836	0.98515
O73	0.65989	0.18532	0.98515
C74	0.72324	0.14368	0.98515
N75	0.67127	0.1146	0.98515
C76	0.65632	0.06081	0.98515
C77	0.54204	0.05351	0.98515
C78	0.48852	0.04181	0.98515
O79	0.48852	0.09055	0.98515
C80	0.44805	0.98946	0.98515
C81	0.61071	0.30026	0.98515
C82	0.64378	0.27737	0.98515
C83	0.77891	0.39553	0.98515
N84	0.81251	0.38056	0.98515
O85	0.73307	0.29102	0.98515
C86	0.86763	0.40967	0.98515
C87	0.89053	0.46564	0.98515
C88	0.86164	0.49832	0.98515
O89	0.81468	0.47457	0.98515
C90	0.85632	0.57956	0.98515
N91	0.8854	0.55667	0.98515
C92	0.93919	0.59551	0.98515
C93	0.94649	0.48853	0.98515
C94	0.95819	0.44671	0.98515
O95	0.90945	0.39797	0.98515
C96	0.01054	0.45859	0.98515
H97	0.44236	0.64902	0.98515
H98	0.33496	0.53651	0.98515
H99	0.59103	0.73953	0.65884
H100	0.61933	0.74169	1.1794
H101	0.5494	0.73635	1.11722
H102	0.60949	0.65579	0.6581
H103	0.57741	0.61255	1.12404
H104	0.63318	0.6877	1.17331
H105	0.42323	0.40231	0.98515
H106	0.35098	0.79334	0.98515
H107	0.46349	0.79846	0.98515
H108	0.26381	0.81117	1.07049

H109	0.26001	0.85706	0.66795
H110	0.25861	0.87396	1.21701
H111	0.35921	0.95757	0.66795
H112	0.37882	0.96263	1.21701
H113	0.30593	0.94383	1.07049
H114	0.59769	0.02092	0.98515
H115	0.20666	0.55764	0.98515
H116	0.20154	0.66504	0.98515
H117	0.15206	0.41283	0.6569
H118	0.12039	0.37853	1.15339
H119	0.18536	0.44888	1.14517
H120	0.05612	0.36223	1.07515
H121	0.04259	0.40117	0.66674
H122	0.03726	0.41652	1.21356
H123	0.97908	0.57677	0.98515
H124	0.55764	0.35098	0.98515
H125	0.66504	0.46349	0.98515
H126	0.44616	0.26331	1.1794
H127	0.41786	0.26115	0.65884
H128	0.37623	0.25797	1.11722
H129	0.36402	0.30852	1.12404
H130	0.39611	0.35176	0.6581
H131	0.41979	0.38367	1.17331
H132	0.57677	0.59769	0.98515
H133	0.64902	0.20666	0.98515
H134	0.53651	0.20154	0.98515
H135	0.73839	0.16227	0.66795
H136	0.74219	0.11638	1.07049
H137	0.73699	0.17917	1.21701
H138	0.68285	0.05327	1.21701
H139	0.66324	0.04822	0.66795
H140	0.60996	0.03448	1.07049
H141	0.40231	0.97908	0.98515
H142	0.79334	0.44236	0.98515
H143	0.79846	0.33496	0.98515
H144	0.81518	0.5517	1.15339
H145	0.84685	0.586	0.6569
H146	0.88015	0.62205	1.14517
H147	0.95194	0.61456	0.66674
H148	0.96548	0.57561	1.07515
H149	0.94661	0.62991	1.21356
H150	0.02092	0.42323	0.98515

Table S2. Fractional atomic coordinates for the unit cell of the BDFamide-Tp COF in staggered stacking mode.

BDFamide-Tp in staggered stacking mode			
Space group: P-1<br>a = 6.6810 Å, b = 26.9603 Å, c = 26.9603 Å, α =60º, β = 90º, γ = 90º			
Atom	x	y	z
C1	-0.75	0.27736	-0.62628
C2	-0.75	0.31781	-0.6104
C3	-0.75	0.43979	-0.74564
N4	-0.75	0.48263	-0.73636
O5	-0.75	0.40678	-0.63785
C6	-0.75	0.53834	-0.77102
C7	-0.75	0.56668	-0.82477
C8	-0.75	0.54598	-0.86279
O9	-0.75	0.49898	-0.84431
C10	-0.75	0.54945	-0.94756
N11	-0.75	0.57555	-0.9168
C12	-0.75	0.63182	-0.94803
C13	-0.75	0.62128	-0.83561
C14	-0.75	0.61932	-0.78821
O15	-0.75	0.56895	-0.74705
C16	-0.75	0.66425	-0.78373
C17	-0.75	0.37372	-0.65108
C18	-0.75	0.3896	-0.70741
C19	-0.75	0.25436	-0.69415
N20	-0.75	0.26364	-0.74627
O21	-0.75	0.36215	-0.76893
C22	-0.75	0.22898	-0.76731
C23	-0.75	0.17523	-0.74191
C24	-0.75	0.13721	-0.68319
O25	-0.75	0.15569	-0.65466
C26	-0.75	0.05244	-0.6019
N27	-0.75	0.0832	-0.65875
C28	-0.75	0.05197	-0.68379
C29	-0.75	0.16439	-0.78567
C30	-0.75	0.21179	-0.8311
O31	-0.75	0.25295	-0.8219
C32	-0.75	0.21627	-0.88052
C33	-0.75	0.34892	-0.72264
C34	-0.75	0.29259	-0.68219
C35	-0.75	0.30585	-0.56021
N36	-0.75	0.25373	-0.51737
O37	-0.75	0.23107	-0.59322
C38	-0.75	0.23269	-0.46166
C39	-0.75	0.25809	-0.43332
C40	-0.75	0.31681	-0.45402
O41	-0.75	0.34534	-0.50102
C42	-0.75	0.3981	-0.45055
N43	-0.75	0.34125	-0.42445
C44	-0.75	0.31621	-0.36818
C45	-0.75	0.21433	-0.37872
C46	-0.75	0.1689	-0.38068
O47	-0.75	0.1781	-0.43105
C48	-0.75	0.11948	-0.33575
C49	-0.25	-0.27736	-0.37372
C50	-0.25	-0.31781	-0.3896

C51	-0.25	-0.43979	-0.25436
N52	-0.25	-0.48263	-0.26364
O53	-0.25	-0.40678	-0.36215
C54	-0.25	-0.53834	-0.22898
C55	-0.25	-0.56668	-0.17523
C56	-0.25	-0.54598	-0.13721
O57	-0.25	-0.49898	-0.15569
C58	-0.25	-0.54945	-0.05244
N59	-0.25	-0.57555	-0.0832
C60	-0.25	-0.63182	-0.05197
C61	-0.25	-0.62128	-0.16439
C62	-0.25	-0.61932	-0.21179
O63	-0.25	-0.56895	-0.25295
C64	-0.25	-0.66425	-0.21627
C65	-0.25	-0.37372	-0.34892
C66	-0.25	-0.3896	-0.29259
C67	-0.25	-0.25436	-0.30585
N68	-0.25	-0.26364	-0.25373
O69	-0.25	-0.36215	-0.23107
C70	-0.25	-0.22898	-0.23269
C71	-0.25	-0.17523	-0.25809
C72	-0.25	-0.13721	-0.31681
O73	-0.25	-0.15569	-0.34534
C74	-0.25	-0.05244	-0.3981
N75	-0.25	-0.0832	-0.34125
C76	-0.25	-0.05197	-0.31621
C77	-0.25	-0.16439	-0.21433
C78	-0.25	-0.21179	-0.1689
O79	-0.25	-0.25295	-0.1781
C80	-0.25	-0.21627	-0.11948
C81	-0.25	-0.34892	-0.27736
C82	-0.25	-0.29259	-0.31781
C83	-0.25	-0.30585	-0.43979
N84	-0.25	-0.25373	-0.48263
O85	-0.25	-0.23107	-0.40678
C86	-0.25	-0.23269	-0.53834
C87	-0.25	-0.25809	-0.56668
C88	-0.25	-0.31681	-0.54598
O89	-0.25	-0.34534	-0.49898
C90	-0.25	-0.3981	-0.54945
N91	-0.25	-0.34125	-0.57555
C92	-0.25	-0.31621	-0.63182
C93	-0.25	-0.21433	-0.62128
C94	-0.25	-0.1689	-0.61932
O95	-0.25	-0.1781	-0.56895
C96	-0.25	-0.11948	-0.66425
C97	-0.25	0.94421	-0.95924
C98	-0.25	-0.01492	-0.94398
C99	-0.25	-0.89404	-0.07962
N100	-0.25	-0.85111	-0.0706
O101	-0.25	0.07414	-0.97217
C102	-0.25	-0.79557	-0.10546
C103	-0.25	-0.76749	-0.15921
C104	-0.25	-0.78857	-0.19695
O105	-0.25	0.16439	-0.17826
C106	-0.25	0.2144	-0.28149
N107	-0.25	0.24074	-0.25097
C108	-0.25	-0.70301	-0.28234

C109	-0.25	-0.71277	-0.17029
C110	-0.25	-0.71463	-0.12293
O111	-0.25	-0.76485	-0.08166
C112	-0.25	-0.66974	-0.11849
C113	-0.25	0.04076	-0.98497
C114	-0.25	-0.94398	-0.0411
C115	-0.25	-0.07962	-0.02634
N116	-0.25	-0.0706	-0.07829
O117	-0.25	0.02783	-0.10197
C118	-0.25	-0.10546	-0.09897
C119	-0.25	-0.15921	-0.0733
C120	-0.25	-0.19695	-0.01448
O121	-0.25	0.82174	-0.98613
C122	-0.25	0.71851	-0.93291
N123	-0.25	0.74903	-0.98977
C124	-0.25	-0.28234	-0.01465
C125	-0.25	-0.17029	-0.11694
C126	-0.25	-0.12293	-0.16244
O127	-0.25	-0.08166	-0.15348
C128	-0.25	-0.11849	-0.21177
C129	-0.25	0.01503	-0.05579
C130	-0.25	-0.0411	-0.01492
C131	-0.25	-0.02634	-0.89404
N132	-0.25	-0.07829	-0.85111
O133	-0.25	0.89803	-0.92586
C134	-0.25	-0.09897	-0.79557
C135	-0.25	-0.0733	-0.76749
C136	-0.25	-0.01448	-0.78857
O137	-0.25	0.01387	-0.83561
C138	-0.25	0.06709	-0.7856
N139	-0.25	0.01023	-0.75926
C140	-0.25	-0.01465	-0.70301
C141	-0.25	-0.11694	-0.71277
C142	-0.25	-0.16244	-0.71463
O143	-0.25	-0.15348	-0.76485
C144	-0.25	-0.21177	-0.66974
C145	-0.75	-0.94421	-0.04076
C146	-0.75	0.01492	-0.05602
C147	-0.75	0.89404	-0.92038
N148	-0.75	0.85111	-0.9294
O149	-0.75	-0.07414	-0.02783
C150	-0.75	0.79557	-0.89454
C151	-0.75	0.76749	-0.84079
C152	-0.75	0.78857	-0.80305
O153	-0.75	-0.16439	-0.82174
C154	-0.75	-0.2144	-0.71851
N155	-0.75	-0.24074	-0.74903
C156	-0.75	0.70301	-0.71766
C157	-0.75	0.71277	-0.82971
C158	-0.75	0.71463	-0.87707
O159	-0.75	0.76485	-0.91834
C160	-0.75	0.66974	-0.88151
C161	-0.75	-0.04076	-0.01503
C162	-0.75	0.94398	-0.9589
C163	-0.75	0.07962	-0.97366
N164	-0.75	0.0706	-0.92171
O165	-0.75	-0.02783	-0.89803
C166	-0.75	0.10546	-0.90103

C167	-0.75	0.15921	-0.9267
C168	-0.75	0.19695	-0.98552
O169	-0.75	-0.82174	-0.01387
C170	-0.75	-0.71851	-0.06709
N171	-0.75	-0.74903	-0.01023
C172	-0.75	0.28234	-0.98535
C173	-0.75	0.17029	-0.88306
C174	-0.75	0.12293	-0.83756
O175	-0.75	0.08166	-0.84652
C176	-0.75	0.11849	-0.78823
C177	-0.75	-0.01503	-0.94421
C178	-0.75	0.0411	-0.98508
C179	-0.75	0.02634	-0.10596
N180	-0.75	0.07829	-0.14889
O181	-0.75	-0.89803	-0.07414
C182	-0.75	0.09897	-0.20443
C183	-0.75	0.0733	-0.23251
C184	-0.75	0.01448	-0.21143
O185	-0.75	-0.01387	-0.16439
C186	-0.75	-0.06709	-0.2144
N187	-0.75	-0.01023	-0.24074
C188	-0.75	0.01465	-0.29699
C189	-0.75	0.11694	-0.28723
C190	-0.75	0.16244	-0.28537
O191	-0.75	0.15348	-0.23515
C192	-0.75	0.21177	-0.33026
H193	-0.75	0.44722	-0.78735
H194	-0.75	0.4754	-0.69575
H195	-0.58253	0.5437	-0.95657
H196	-0.83782	0.5744	-0.98563
H197	-0.82964	0.50985	-0.92451
H198	-0.58249	0.64667	-0.95563
H199	-0.83634	0.6489	-0.92631
H200	-0.83117	0.6443	-0.98681
H201	-0.75	0.6606	-0.7435
H202	-0.75	0.21265	-0.65987
H203	-0.75	0.30425	-0.77965
H204	-0.79345	0.07852	-0.58517
H205	-0.58815	0.03531	-0.58759
H206	-0.8684	0.01945	-0.58848
H207	-0.5834	0.04743	-0.69434
H208	-0.84859	0.07199	-0.72077
H209	-0.818	0.01183	-0.65603
H210	-0.75	0.2565	-0.9171
H211	-0.75	0.34013	-0.55278
H212	-0.75	0.22035	-0.5246
H213	-0.5832	0.41273	-0.45386
H214	-0.84697	0.4113	-0.42695
H215	-0.81983	0.41472	-0.49124
H216	-0.79352	0.34592	-0.35594
H217	-0.58813	0.29993	-0.35269
H218	-0.86834	0.28301	-0.35149
H219	-0.75	0.0829	-0.3394
H220	-0.25	-0.44722	-0.21265
H221	-0.25	-0.4754	-0.30425
H222	-0.33782	-0.5109	-0.07449
H223	-0.08253	-0.5416	-0.04542
H224	-0.32964	-0.57545	-0.01337

H225	-0.33634	-0.64435	-0.01382
H226	-0.08249	-0.64658	-0.04313
H227	-0.33117	-0.64895	-0.07431
H228	-0.25	-0.6606	-0.2565
H229	-0.25	-0.21265	-0.34013
H230	-0.25	-0.30425	-0.22035
H231	-0.08815	-0.05355	-0.41343
H232	-0.29345	-0.01033	-0.41101
H233	-0.3684	-0.0694	-0.41432
H234	-0.34859	-0.01551	-0.34001
H235	-0.0834	-0.04007	-0.31358
H236	-0.318	-0.07568	-0.27527
H237	-0.25	-0.2565	-0.0829
H238	-0.25	-0.34013	-0.44722
H239	-0.25	-0.22035	-0.4754
H240	-0.34697	-0.41454	-0.51225
H241	-0.0832	-0.41311	-0.53916
H242	-0.31983	-0.41112	-0.57654
H243	-0.08813	-0.31931	-0.64594
H244	-0.29352	-0.27332	-0.64919
H245	-0.36834	-0.33623	-0.64475
H246	-0.25	-0.0829	-0.6606
H247	-0.25	-0.88681	-0.12126
H248	-0.25	-0.85815	-0.03006
H249	-0.08255	0.2087	-0.29055
H250	-0.33827	0.23914	-0.31954
H251	-0.32918	0.17476	-0.25824
H252	-0.08249	-0.68817	-0.29004
H253	-0.33605	-0.68582	-0.26068
H254	-0.33146	-0.69065	-0.32105
H255	-0.25	-0.67337	-0.07827
H256	-0.25	-0.12126	0.00807
H257	-0.25	-0.03006	-0.11179
H258	-0.29343	0.74476	-0.91637
H259	-0.08816	0.70141	-0.91855
H260	-0.36841	0.68551	-0.91935
H261	-0.08344	-0.28683	-0.02526
H262	-0.34892	-0.26246	-0.05158
H263	-0.31764	-0.32248	0.01324
H264	-0.25	-0.07827	-0.24837
H265	-0.25	0.00807	-0.88681
H266	-0.25	-0.11179	-0.85815
H267	-0.08315	0.08175	-0.78912
H268	-0.34649	0.08045	-0.76206
H269	-0.32036	0.08352	-0.82622
H270	-0.29354	0.01515	-0.6909
H271	-0.08813	-0.0309	-0.68749
H272	-0.36833	-0.04785	-0.68623
H273	-0.25	-0.24837	-0.67337
H274	-0.75	0.88681	-0.87874
H275	-0.75	0.85815	-0.96994
H276	-0.83827	-0.17593	-0.74065
H277	-0.58255	-0.20637	-0.71167
H278	-0.82918	-0.24031	-0.67935
H279	-0.83605	0.6906	-0.6795
H280	-0.58249	0.68825	-0.70886
H281	-0.83146	0.68577	-0.73987
H282	-0.75	0.67337	-0.92173

H283	-0.75	0.12126	-1.00807
H284	-0.75	0.03006	-0.88821
H285	-0.58816	-0.71971	-0.08237
H286	-0.79343	-0.67636	-0.08019
H287	-0.86841	-0.73561	-0.08317
H288	-0.84892	0.31872	-1.00919
H289	-0.58344	0.29435	-0.98286
H290	-0.81764	0.2587	-0.94436
H291	-0.75	0.07827	-0.75163
H292	-0.75	-0.00807	-0.11319
H293	-0.75	0.11179	-0.14185
H294	-0.84649	-0.08336	-0.17713
H295	-0.58315	-0.08207	-0.20419
H296	-0.82036	-0.08029	-0.24129
H297	-0.58813	0.0115	-0.31107
H298	-0.79354	0.05755	-0.31448
H299	-0.86833	-0.00545	-0.30982
H300	-0.75	0.24837	-0.32663

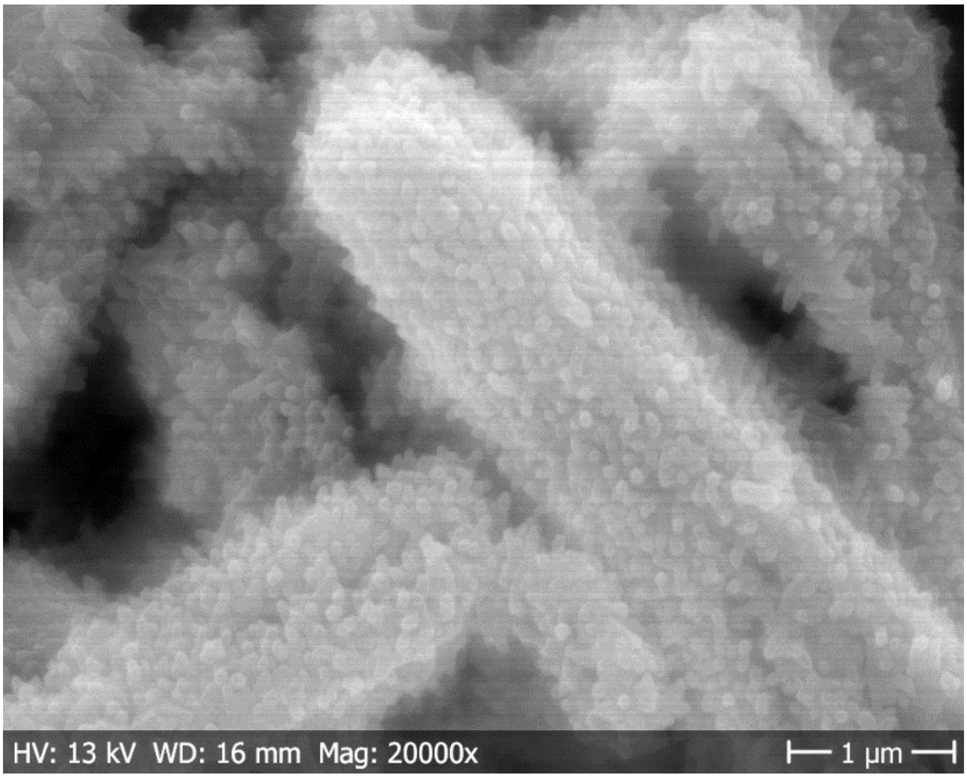

Figure S5. SEM image of BDFamide-Tp COF powder under × 20,000 magnification.

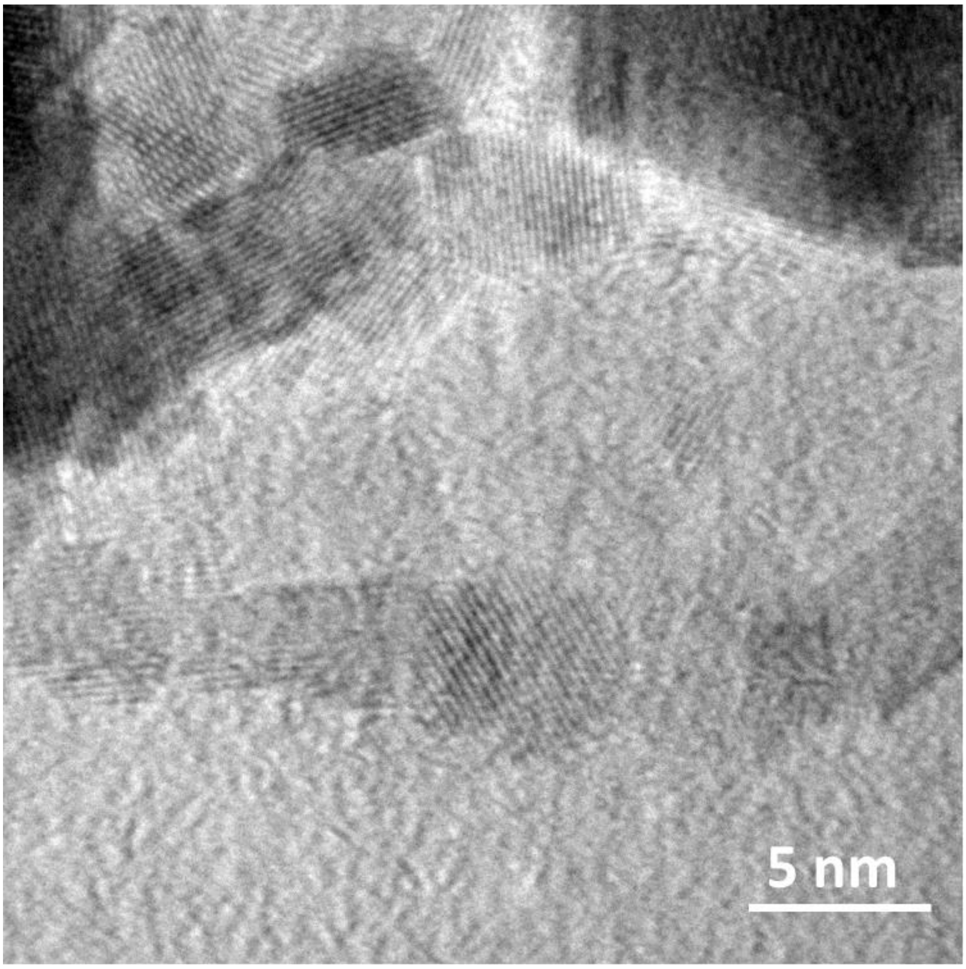

Figure S6. TEM image of BDFamide-Tp COF showing a polycrystalline state.

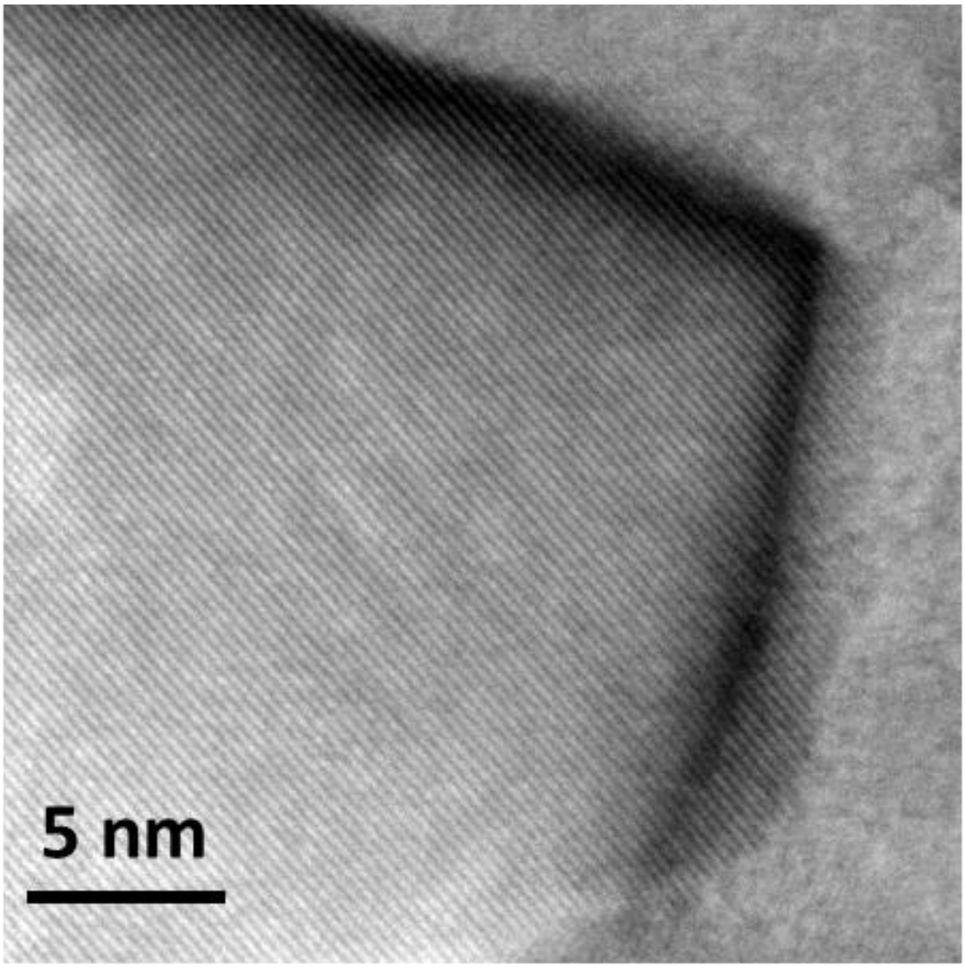

Figure S7. Additional HRTEM of a BDFamide-Tp COF.

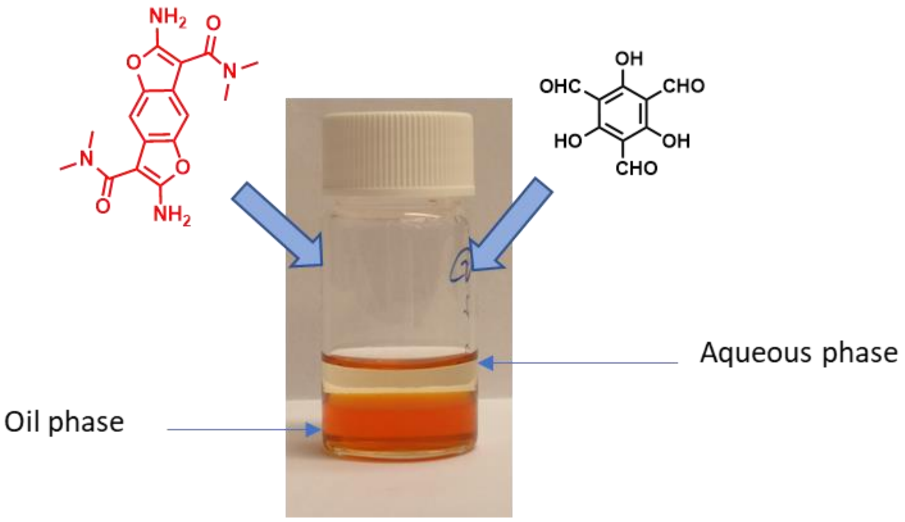

Figure S8. Illustration of the interfacial synthesis of the BDFamide-Tp 2D COF film. The liquid-liquid system is formed by mixing an NMP solution of BDF-dicarboxamide (2 mL), a chloroform solution containing Tp (2 mL) and aqueous acetic acid (3 mL).

Table S3. Optimization of the interfacial synthesis for high quality BDFamide-Tp COF film.

**Number**	**BDF-dicarboxamide (NMP solution)[a]/ mL**	**Tp ($CHCl_3$ solution)[b]/ mL**	**Acetic acid (6M, aq)/ mL**	**Area of interface /cm^2**	**Product at the interface**
**1**	1	1	3	4.91	powder paticles
**2**	1	1	6	4.91	more fluffy powders
**3**	1	1	9	4.91	more fluffy powders
**4**	1.5	1.5	3	4.91	flakes
**5**	1.5	1.5	6	4.91	**film** with flakes
**6**	1.5	1.5	9	4.91	**film** with particles
**7**	2	2	3	4.91	smooth **film**
**8**	2	2	6	4.91	coarse **film**
**9**	2	2	9	4.91	coarse **film** with particles

[a] concentration: 1.89×10^{-3} M; **[b]** concentration: 1.25×10^{-3} M

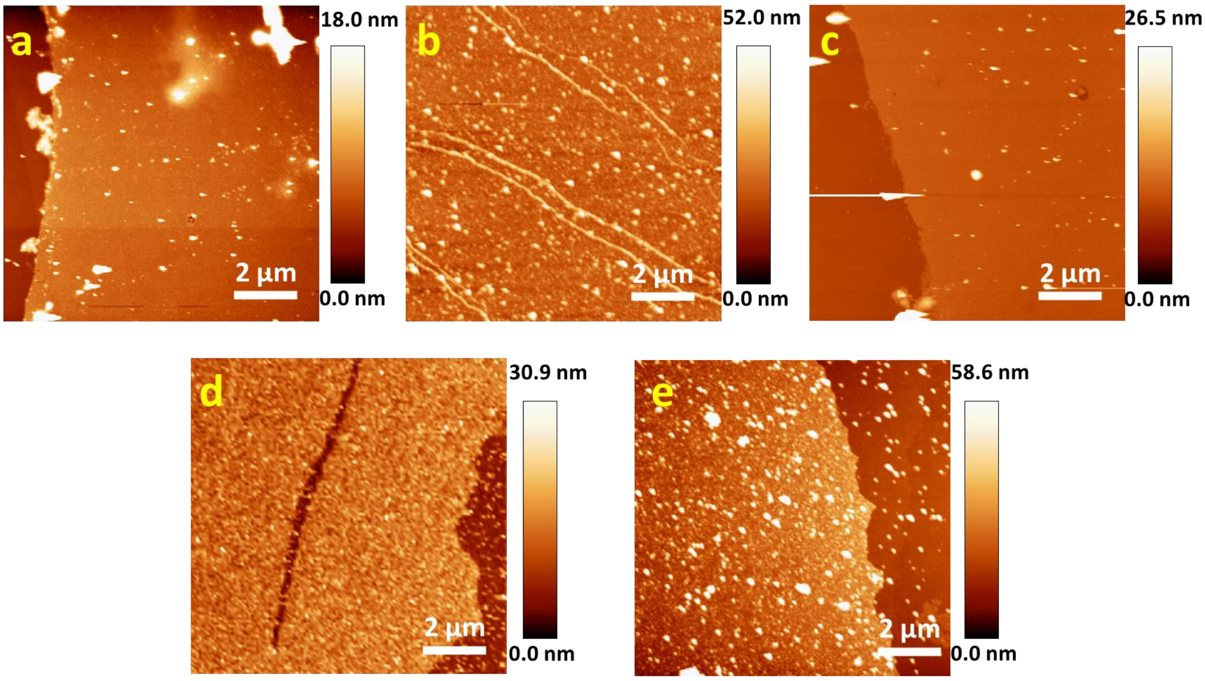

Figure S9. AFM height images of BDFamide-Tp COF films prepared via interfacial synthesis, for synthetic condition optimization towards a high quality film. The respective synthetic conditions are shown in Table S3. (a) corresponds to number 5, (b) corresponds to number 6, (c) corresponds to number 7, (d) corresponds to number 8, (e) corresponds to number 9.

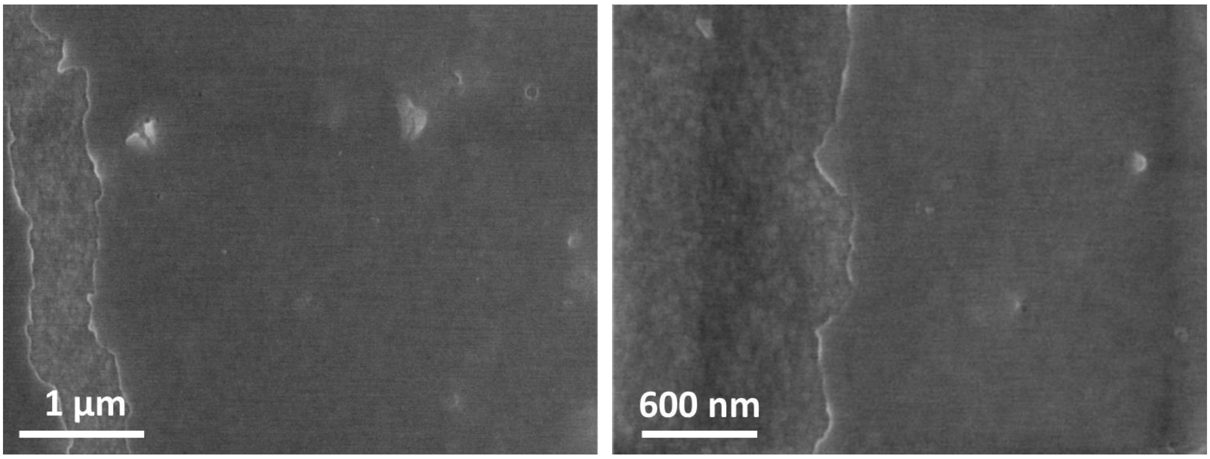

Figure S10. SEM images of a BDFamide-Tp film at high magnification.

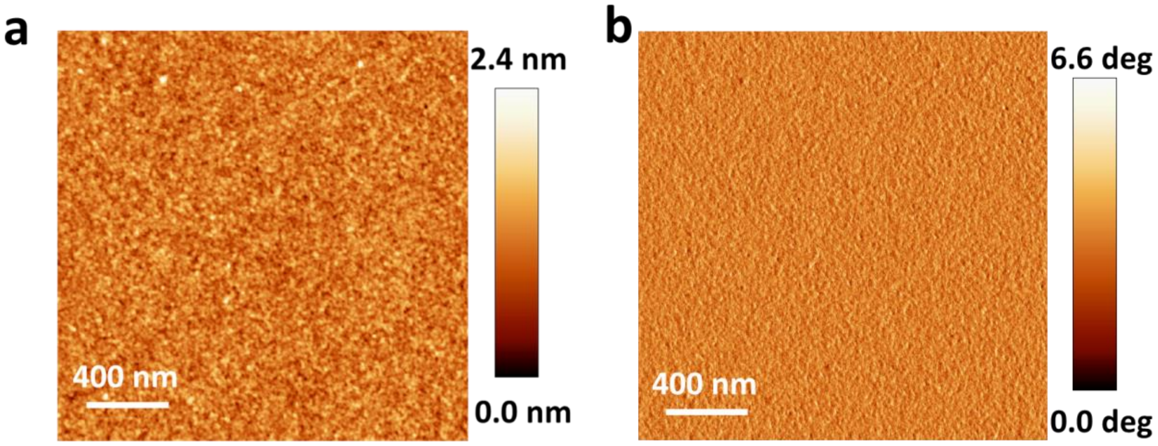

Figure S11. AFM height image (a) and phase image (b) of a BDFamide-TP COF film synthesized using the optimized condition. The evaluated area in (a) gives an RMS roughness of 0.35 nm.

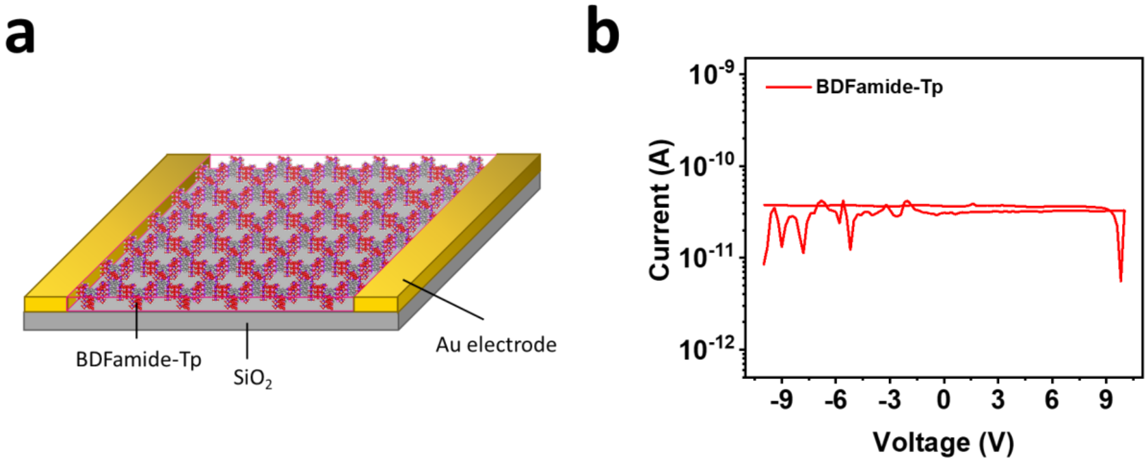

Figure S12. (a) Illustration of device structure for conductivity measurements of BDFamide-Tp films. (b) I-V curve of the device in a, showing poor conductivity of BDFamide-Tp.

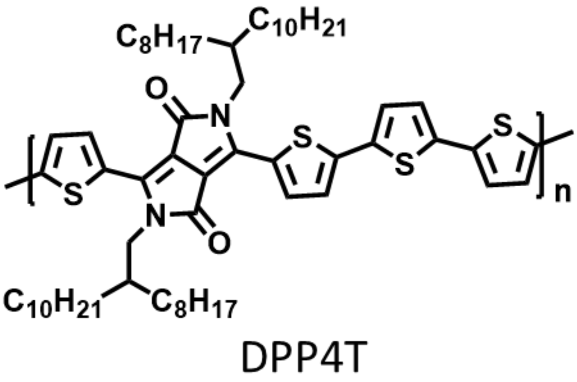

Figure S13. Chemical structure of DPP4T.

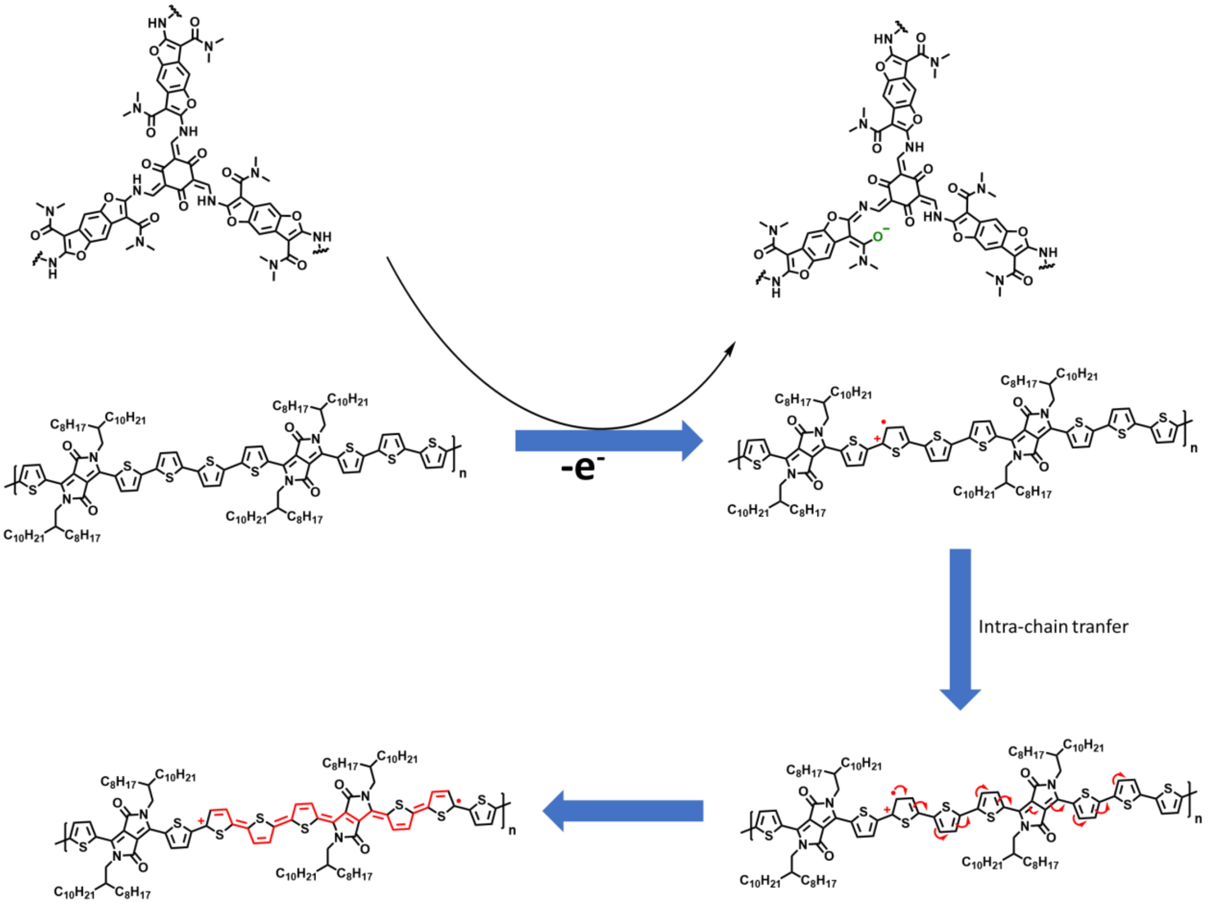

Figure S14. Proposed mechanism of the interfacial doping effect in the BDFamide-Tp COF/DPP4T system.

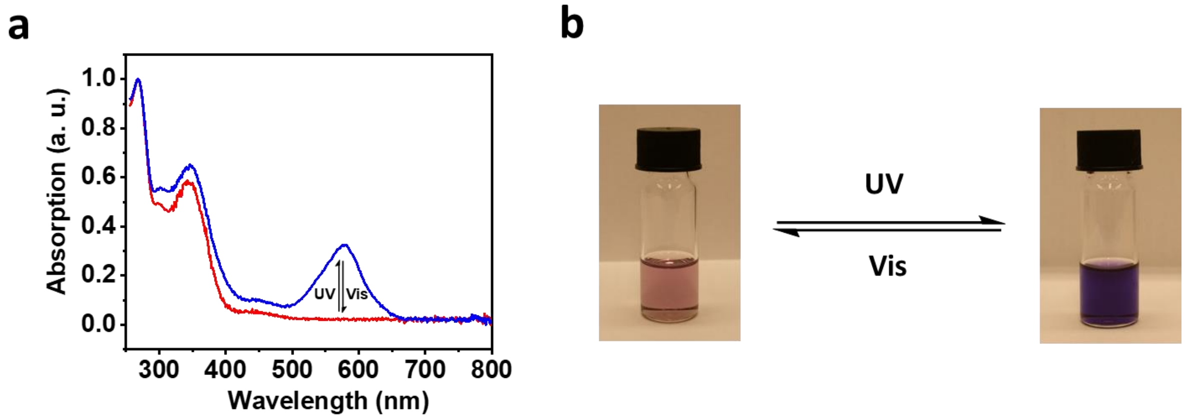

Figure S15. The reversible photoswitching property of the spiropyran-merocyanine couple under UV/Vis, as displayed by UV-Vis spectra (a) and image of the solution (b). The solvent was chloroform in both cases.

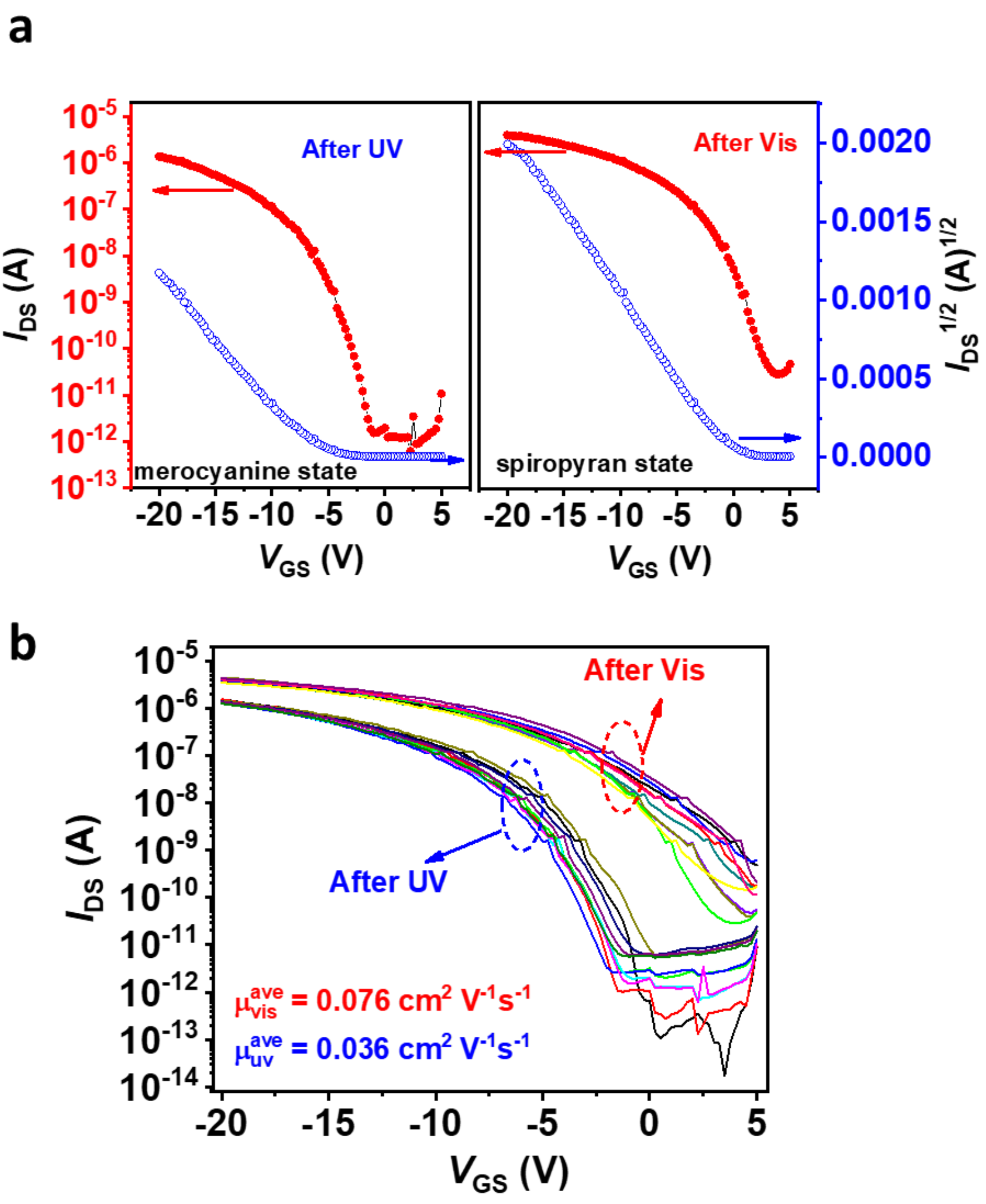

Figure S16. The mobilities of a semiconductor (DPP4T) in presence of the neutral spiropyran and the zwitterionic merocyanine. (a) Transfer curves of the field-effect transistor (FET) with DPP4T blended with spiropyran as the semiconducting material. Left shows the transfer curves after UV exposure, which promotes the zwitterionic merocyanine in the semiconductor. Right shows the transfer curves after Vis exposure, which promotes the neutral spiropyran in the semiconductor. (b) Transfer curves during 10 cycles of switching between spiropyran and merocyanine. These were used for calculating the average mobilities. The FET is in bottom-gate-bottom-contact configuration with a structure of DPP4T(blended with spiropyran)/Au/SiO2/Si. The device channel was 50 µm in length and 1400 µm in width. The applied gate voltage was -20 V.

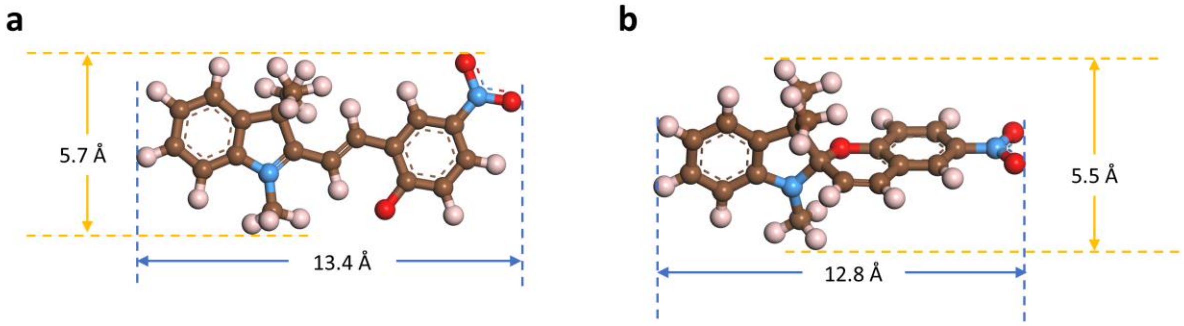

Figure S17. Simulated structure of merocyanine (a) and spiropyran (b), showing molecular sizes in different directions. The simulated sizes are shorter than the pore width of 1.35 nm in the BDFamide-Tp, illustrating on the theoretical possibility of encapsulation.

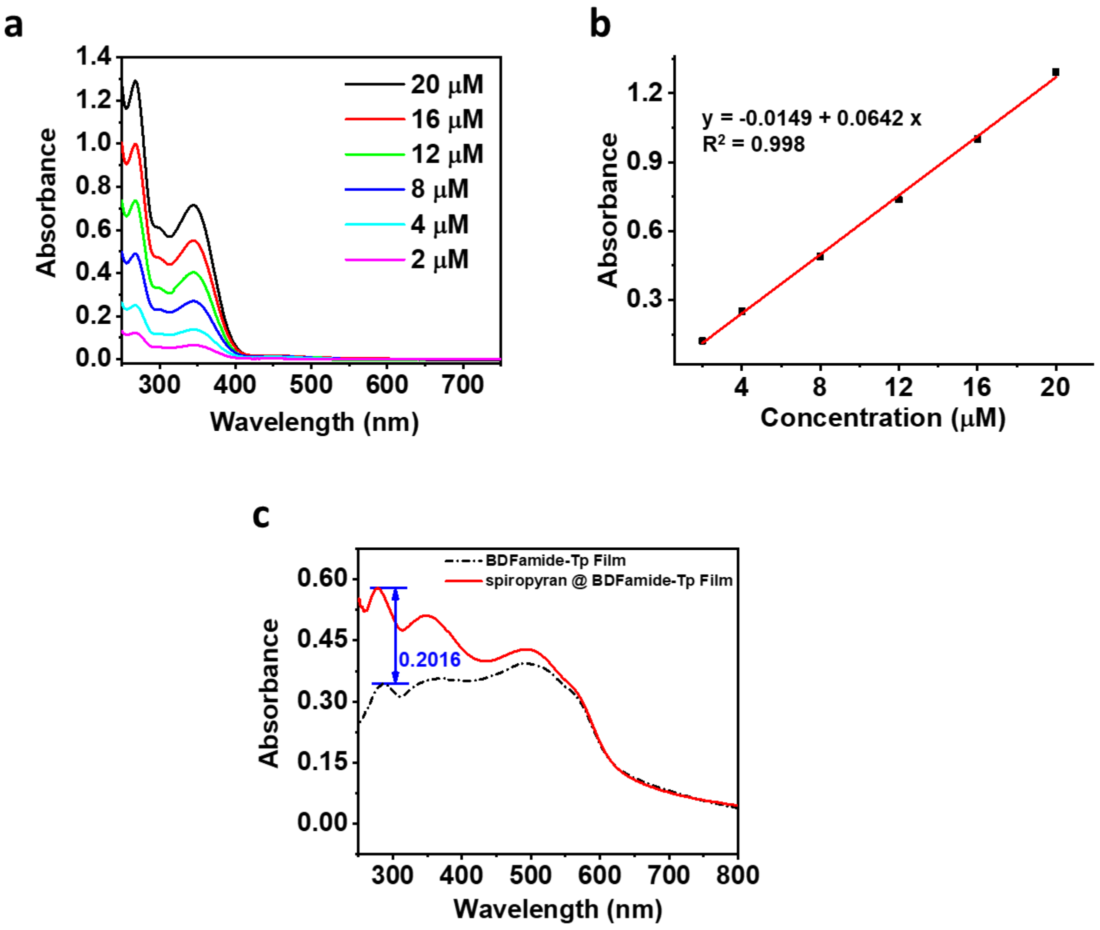

Figure S18. Determination of the content of spiropyran encapsulated in the BDFamide COF film. (a) UV-Vis spectra of spiropyran solutions (in chloroform) at different concentrations. (b) The absorbance at 269 nm of spiropyran with respect to concentration. The derivative of the curve was used to calculate the molar extinction coefficient to 6.3 x 10^4 M^{-1} cm^{-1} (c) UV-Vis spectra of a 495 nm thick BDFamide COF film before and after spiropyran encapsulation. According to the Lambert-Beer's law, the concentration of spiropyran encapsulated in the COF film is 0.065 mol/L.

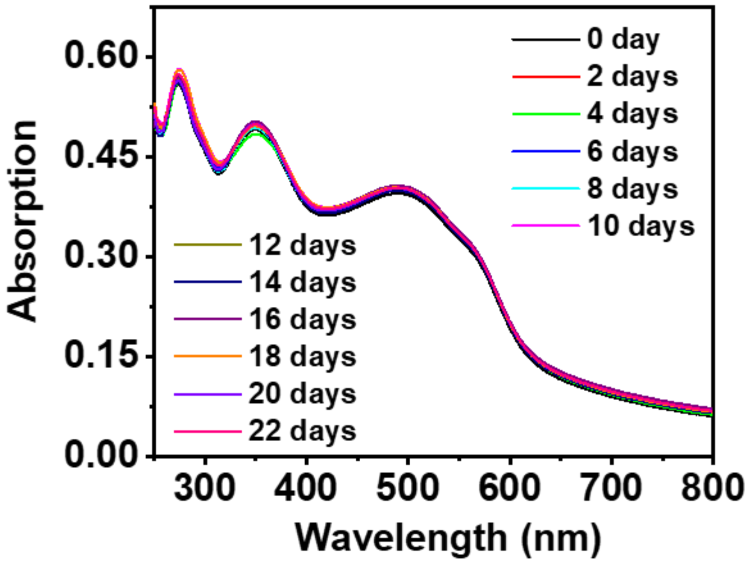

Figure S19. UV-vis spectra of a spiropyran encapsulated BDFamide COF film, recorded regularly for 22 days. The film was stored in ambient conditions, and before each measurement the film was exposed to white light for 5 min to ensure that the photoswitch was in the spiropyran state.

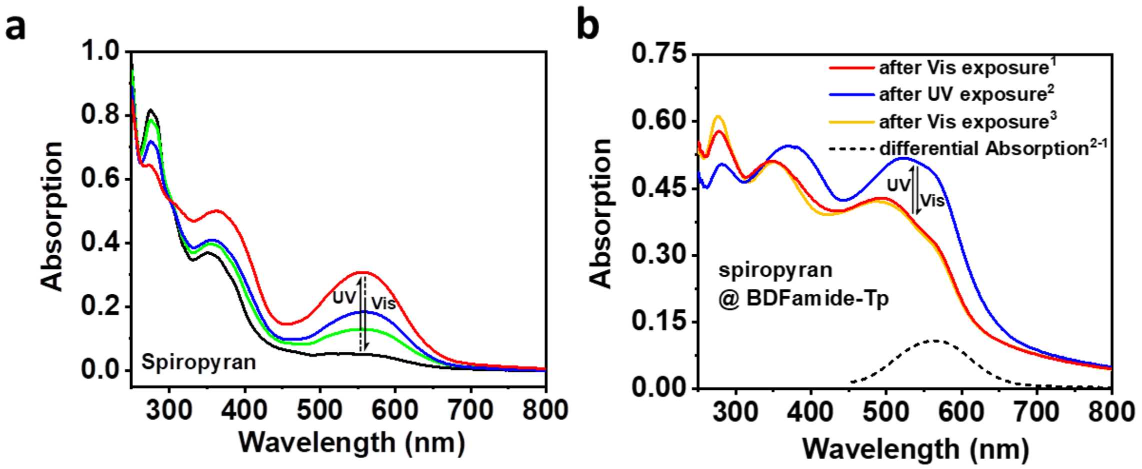

Figure S20. (a) The UV-Vis spectral change of a neat spiropyran film under UV/Vis exposure. (b) The UV-Vis spectral change of a BDFamide-Tp COF film with encapsulated spiropyran after UV/Vis exposure.

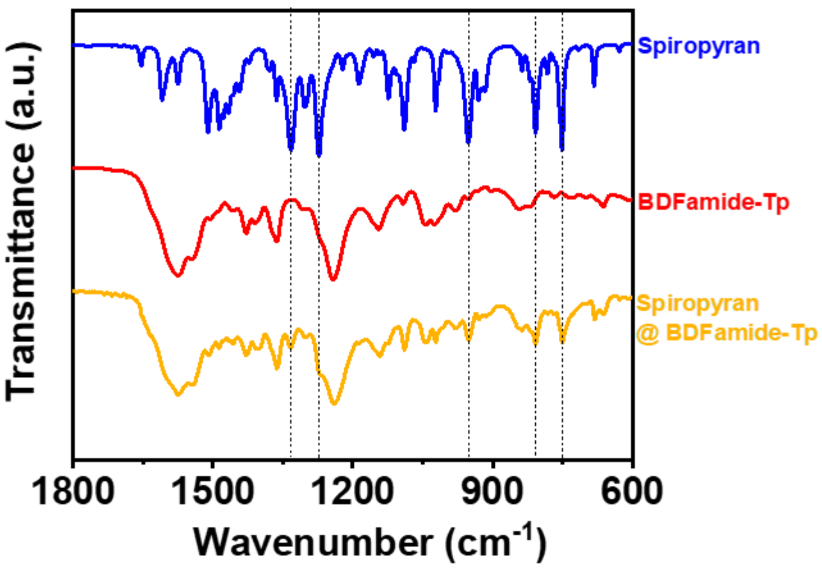

Figure S21. FTIR spectra of spiropyran, BDFamide-Tp COF and BDFamide-Tp after spiropyran encapsulation.

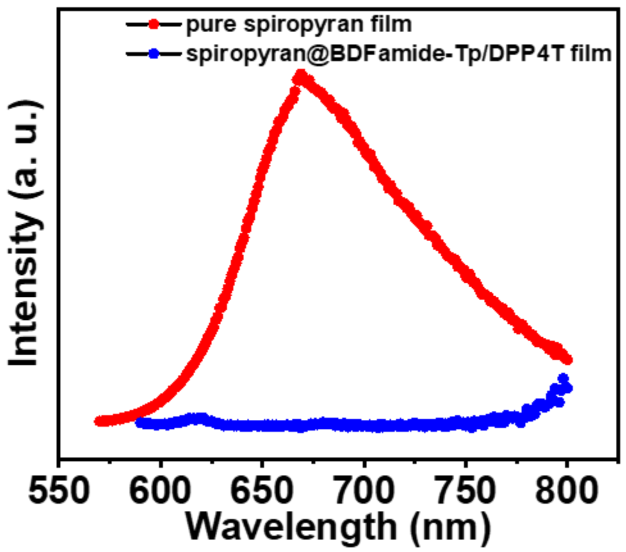

Figure S22. Emission spectra of a pure spiropyran film (exposed to UV light for 5 min) and a spiropyran encapsulated in BDFamide-TP film spincoated with DPP4T (the active layer in the photoswitchable device, exposed to UV light for 5 min). Excitation wavelength is 550 nm.

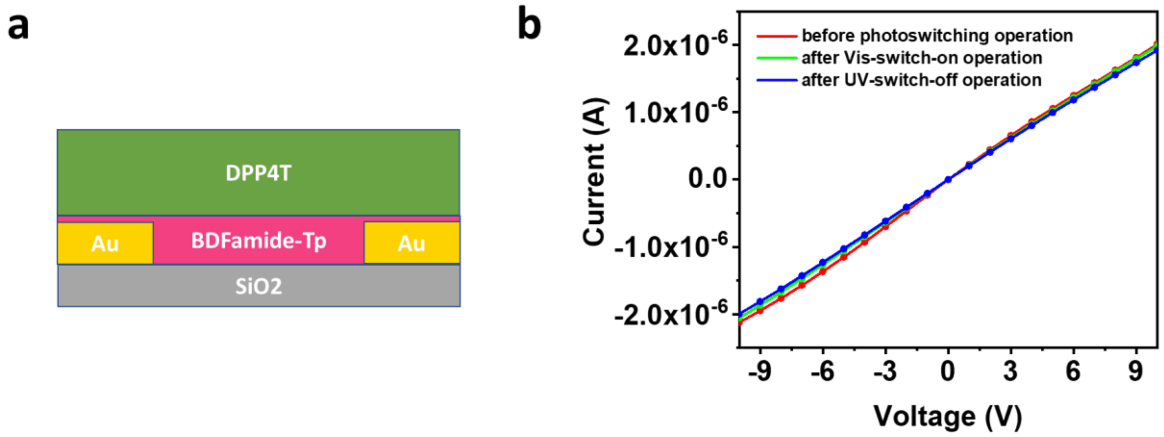

Figure S23. Control experiment for photo-stimulus-responsivity. (a) Illustration of device structure with DPP4T/BDFamide-Tp as active layer but without encapsulation of spiropyran. (b) I-V curve of pristine device, and I-V curves of the device after 6 min UV illumination and after 8 min visible light illumination.

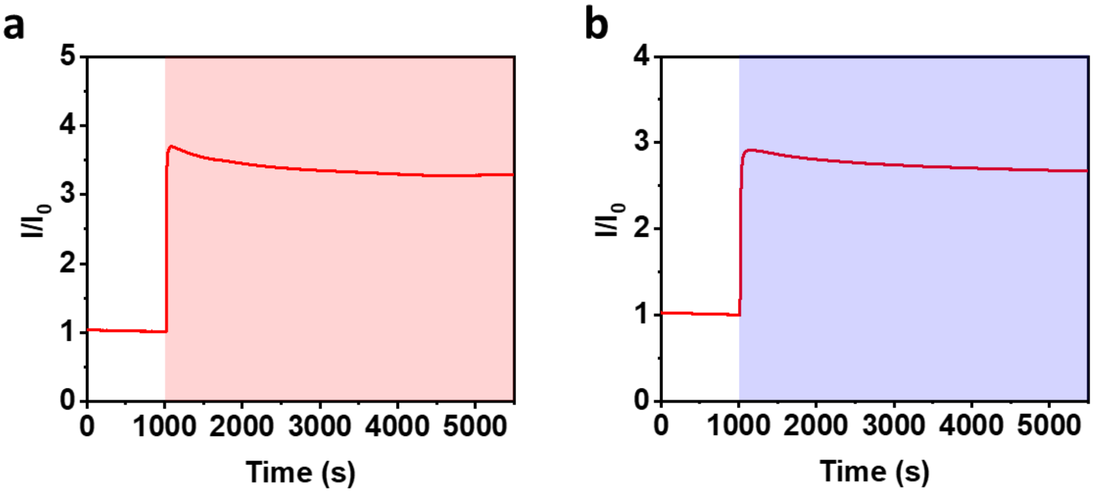

Figure S24. Constant recording of output current of the device shown in Figure S14 (a) upon continuous illumination of visible light (a) and UV light (b).

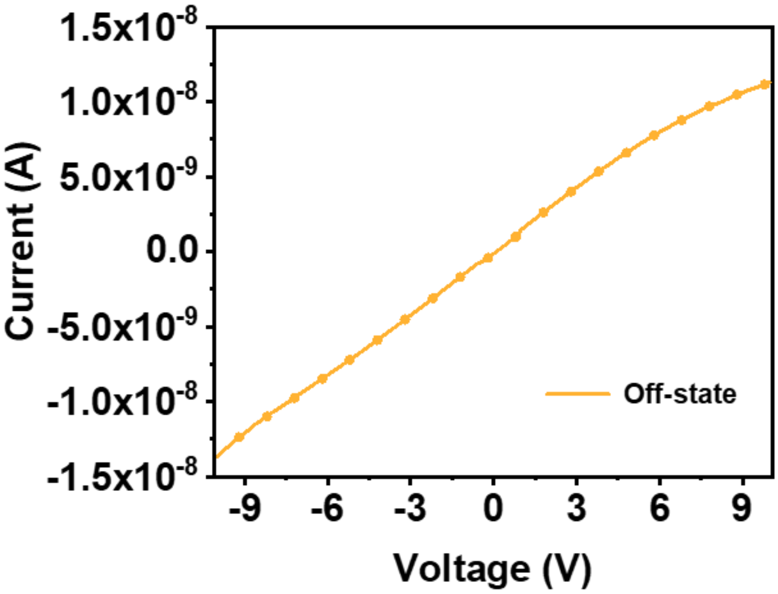

Figure S25. I-V curve of the photoswitchable device after 8 min of UV-illumination (rescaled plot of the Off-state in Figure 5h).

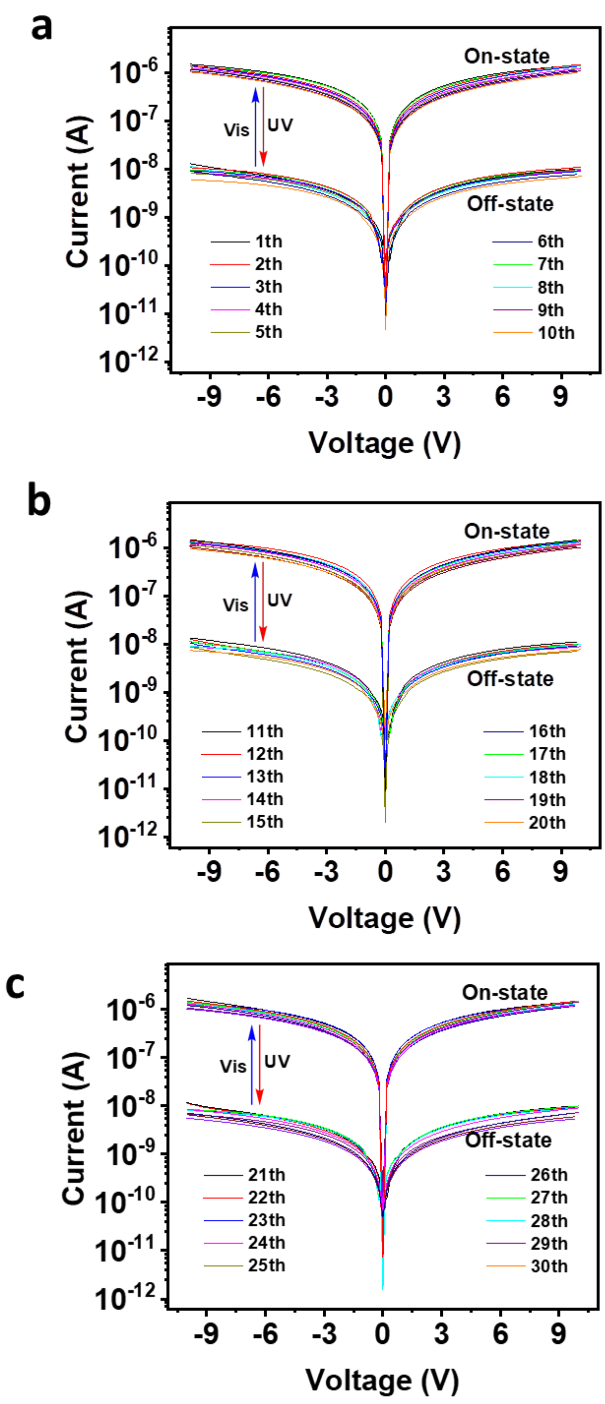

Figure S26. I-V curves (in logarithm) of photo-stimulus-responsive device in on- and off-state switched for 30 cycles. (a) 1st-10th scan cycle of on-/off state. (b) 11th-20th scan cycle of on-/off state. (c) 20st-30th scan cycle of on-/off state.

References:

1. Yao, J.; Yu, C.; Liu, Z.; Luo, H.; Yang, Y.; Zhang, G.; Zhang, D. Significant Improvement of Semiconducting Performance of the Diketopyrrolopyrrole-Quaterthiophene Conjugated Polymer through Side-Chain Engineering via Hydrogen-Bonding. J. Am. Chem. Soc. 2016, 138, 173– 185.
